# Supplementary figures and images for: Long-term neurodevelopmental outcome in children born after vacuum-assisted delivery compared with second-stage caesarean delivery and spontaneous vaginal delivery: a cohort study
Source: BMJ Paediatr Open. 2023 Oct 17;7(1):e002048. doi: 10.1136/bmjpo-2023-002048 (PMC10582903; doi:10.1136/bmjpo-2023-002048)

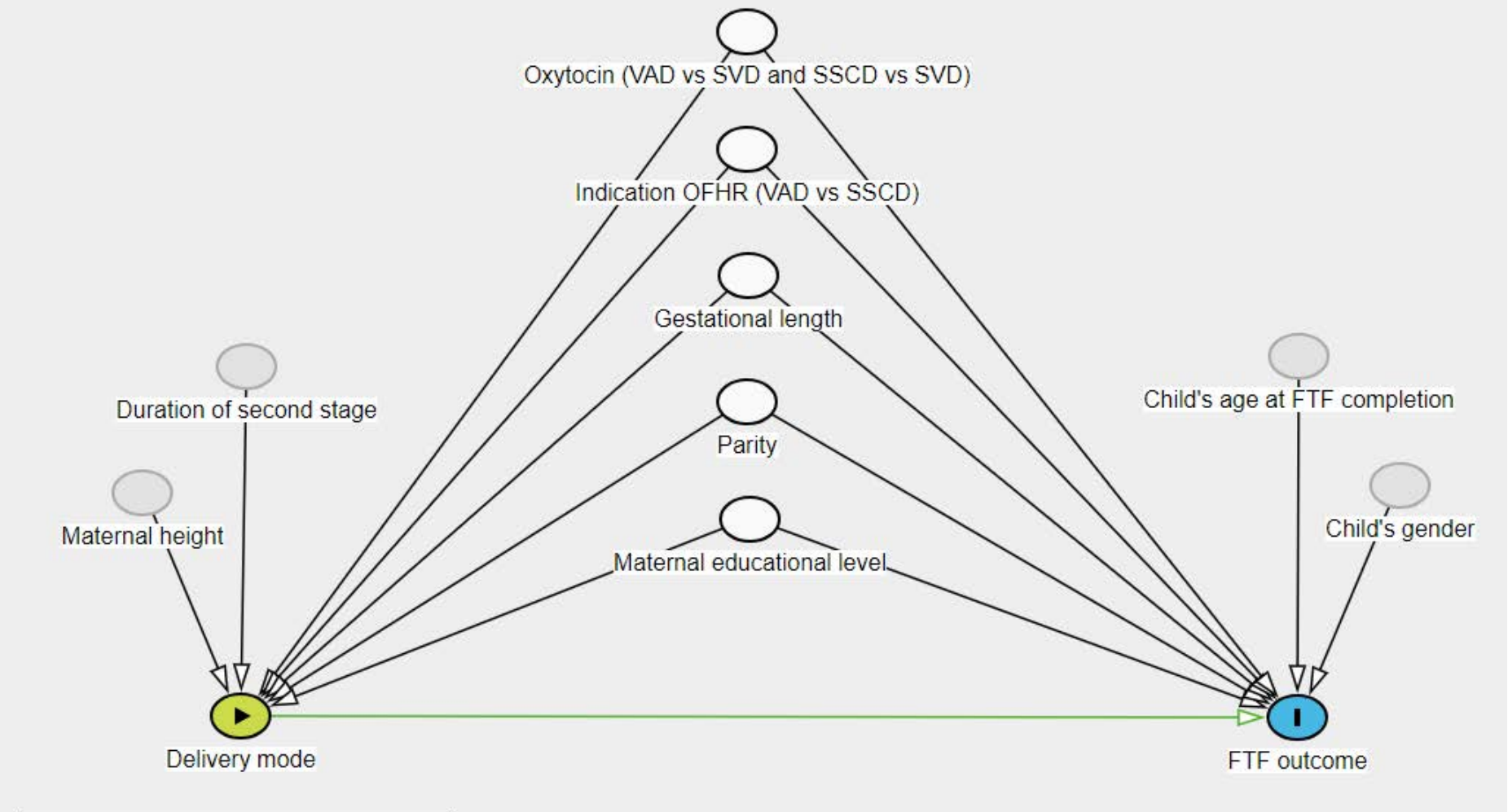

Figure S1: Directed Acyclic Graph (DAG)

Supplement: Supplementary data [file bmjpo-2023-002048supp001.pdf]
