# Supplementary material for: Long-term neurodevelopmental outcome in children born after vacuum-assisted delivery compared with second-stage caesarean delivery and spontaneous vaginal delivery: a cohort study
Source: BMJ Paediatr Open. 2023 Oct 17;7(1):e002048. doi: 10.1136/bmjpo-2023-002048 (PMC10582903; doi:10.1136/bmjpo-2023-002048)
Supplement: Supplementary data [file bmjpo-2023-002048supp002.pdf]

Table S1: Descriptive analysis of respondents and non-respondents in the vacuum-assisted delivery group

	FTF (n =253)	No FTF (n =187)	p-value
**Maternal characteristics**			
BMI (kg/m^2)[3]	23 (16-50)[§]	23 (17-39)[†]	0.82[6]
Nulliparous[1]	208 (82%)	146 (78%)	0.28[4]
Previous caesarean[1]	23 (9%)	20 (11%)	0.58[4]
Gestational length, days[2]	281±8	282±9	0.30[5]
**Delivery characteristics**			
First stage of labour, hours[2]	9±5	9±5	0.56[5]
Second stage of labour, hours[2]	2±2	2±2	0.22[5]
Epidural[1]	175 (72%)	135 (78%)	0.22[4]
Oxytocin[1]	242 (96%)	172 (92%)	0.11[4]
Indication OFHR[1]	108 (43%)	89 (48%)	0.31[4]
Station Mid[1]	148 (59%)	133 (71%)	<0.05[4*]
Number of pulls, n[3]	4 (1-10)	4 (1-13)	0.4[6]
Time during vacuum extraction, min[3]	7 (1-31)	7 (1-29)	0.94[6]
Position OAP[1]	221 (87%)	159 (85%)	0.48[4]
Subjectively heavy extraction[1]	58 (25%)	48 (28%)	0.44[4]
Failed vacuum extraction[1]	34 (13%)	33 (18%)	0.23[4]
Cup detachment[1]	39 (15%)	29 (16%)	0.98[4]
Shoulder dystocia[1]	11 (4%)	13 (7%)	0.23[4]
**Perinatal characteristics**			
Birth weight, g[2]	3668 ± 447	3641 ± 455	0.54[5]
Gender male[1]	138 (55%)	118 (63%)	0.07[4]
pH<7.00[1]	4 (2%)[‡]	0[ǂ]	0.09[4]
Apgar<7 at 5 min[1]	9 (4%)	9 (5%)	0.51[4]
Severe outcome[1] (ICH, HIE, seizures, subgaleal haematoma)	4 (2%)	5 (3%)	0.42[4]

*Statistically significant at p-value <0.05. [1]n (%), [2]mean±sd, [3]median (min–max), [4]Chi² test, [5]Student T-test, [6]Wilcoxon rank sum test. [§]missing data 11%, [†]missing data 10%, [‡]missing data 18%, [ǂ]missing data 19%. FTF: Five- to Fifteen questionnaire. BMI: body mass index, OFHR: ominous foetal heart rate, OAP: occipital-anterior position, ICH: intracranial haemorrhage, HIE: hypoxic ischemic encephalopathy
