# Supplementary material for: Long-term neurodevelopmental outcome in children born after vacuum-assisted delivery compared with second-stage caesarean delivery and spontaneous vaginal delivery: a cohort study
Source: BMJ Paediatr Open. 2023 Oct 17;7(1):e002048. doi: 10.1136/bmjpo-2023-002048 (PMC10582903; doi:10.1136/bmjpo-2023-002048)
Supplement: Author's manuscript [file bmjpo-2023-002048.draft_revisions.pdf]

BMJ Paediatrics Open

BMJ Paediatrics Open is committed to open peer review. As part of this commitment we make the peer review history of every article we publish publicly available.

When an article is published we post the peer reviewers' comments and the authors' responses online. We also post the versions of the paper that were used during peer review. These are the versions that the peer review comments apply to.

The versions of the paper that follow are the versions that were submitted during the peer review process. They are not the versions of record or the final published versions. They should not be cited or distributed as the published version of this manuscript.

BMJ Paediatrics Open is an open access journal and the full, final, typeset and author-corrected version of record of the manuscript is available on our site with no access controls, subscription charges or pay-per-view fees (http://bmjpaedsopen.bmj.com).

If you have any questions on BMJ Paediatrics Open's open peer review process please email info.bmjpo@bmj.com

BMJ Paediatrics Open

Long–term neurodevelopmental outcome in children born after vacuum-assisted delivery -an observational cohort study

Journal:	*BMJ Paediatrics Open*
Manuscript ID	bmjpo-2023-002048
Article Type:	Original research
Date Submitted by the Author:	04-May-2023
Complete List of Authors:	Romero, Stefhanie; Karolinska Institute Department of Clinical Science Intervention and Technology, Clinical science, intervention and technology; Karolinska University Hospital, Pregnancy Care and Delivery<br>Lindström, Katarina; Karolinska Institute Department of Clinical Science Intervention and Technology, Clinical Science Intervention and Technology; Karolinska University Hospital, Neuropaediatrics<br>Listermar, Johanna; Karolinska Institute<br>Westgren, Magnus; Karolinska Institute Department of Clinical Science Intervention and Technology, Clinical Science Intervention and Technology<br>Ajne, Gunilla; Karolinska Institute Department of Clinical Science Intervention and Technology, Clinical Science Intervention and Technology; Karolinska University Hospital, Pregnancy Care and Delivery
Keywords:	Psychology, Pathology, Neonatology, Epidemiology, Adolescent Health

SCHOLARONE™
Manuscripts

I, the Submitting Author has the right to grant and does grant on behalf of all authors of the Work (as defined in the below author licence), an exclusive licence and/or a non-exclusive licence for contributions from authors who are: i) UK Crown employees; ii) where BMJ has agreed a CC-BY licence shall apply, and/or iii) in accordance with the terms applicable for US Federal Government officers or employees acting as part of their official duties; on a worldwide, perpetual, irrevocable, royalty-free basis to BMJ Publishing Group Ltd ("BMJ") its licensees and where the relevant Journal is co-owned by BMJ to the co-owners of the Journal, to publish the Work in this journal and any other BMJ products and to exploit all rights, as set out in our licence.

The Submitting Author accepts and understands that any supply made under these terms is made by BMJ to the Submitting Author unless you are acting as an employee on behalf of your employer or a postgraduate student of an affiliated institution which is paying any applicable article publishing charge ("APC") for Open Access articles. Where the Submitting Author wishes to make the Work available on an Open Access basis (and intends to pay the relevant APC), the terms of reuse of such Open Access shall be governed by a Creative Commons licence – details of these licences and which Creative Commons licence will apply to this Work are set out in our licence referred to above.

Other than as permitted in any relevant BMJ Author's Self Archiving Policies, I confirm this Work has not been accepted for publication elsewhere, is not being considered for publication elsewhere and does not duplicate material already published. I confirm all authors consent to publication of this Work and authorise the granting of this licence.

Confidential: For Review Only

Long–term neurodevelopmental outcome in children born after vacuum-assisted delivery -an observational cohort study

Stefhanie Romero[1,2], MD PhD, Katarina Lindström[3,4], MD PhD, Johanna Listermar, Medical student[5], Magnus Westgren[1], MD PhD, Gunilla Ajne[1,2], MD PhD

[1]Division of Obstetrics and Gynecology, CLINTEC, Karolinska Institutet, Stockholm, 171 77, Sweden
[2] Pregnancy Care & Delivery, Karolinska University Hospital, Stockholm, 141 86, Sweden,
[3]Division of Pediatrics, CLINTEC, Karolinska Institutet, Stockholm, 171 77, Sweden
[4]Neuropediatrics, Karolinska University Hospital, Stockholm, 141 86, Sweden
[5]Karolinska Institutet, Stockholm, 171 77, Sweden
Correspondence to: Stefhanie Romero, Pregnancy Care & Delivery, B76, Karolinska University Hospital, Stockholm, 141 86, Sweden. stefhanie.romero@ki.se.

Confidential: For Review Only

Introduction

The vacuum extractor was developed as an alternative to the obstetric forceps and emergency cesarean delivery (ECD) to be used in cases of dystocia or fetal distress during the second stage of labor.[1] Prevalence of vacuum-assisted delivery (VAD) varies greatly between different countries, representing up to 3% of the assisted vaginal births in the USA[2], and 5–10% in Europe, Canada, and Australia/New Zealand.[3-7]
Despite the fact that serious fetal complications are rare, VAD appears to have a higher rate of intracranial hemorrhage (ICH)[8] and subgaleal hematomas[9] compared to spontaneous vaginal delivery (SVD).[10, 11] When comparing VAD to other instrumental delivery modes, the results are not consistent. Several studies compare VAD to ECD at the second stage of labor; some show no differences in severe neonatal outcomes,[12, 13] while others provide evidence indicating an increased risk of birth trauma in the VAD group.[14, 15]

VAD is classified according to fetal head station in the birth canal into the mid, low and outlet stations, the last of which is considered a low risk VAD.[16, 17] Previous research has not always taken this stratification into account, which could be one of the reasons behind divergent results. Considering the frequent use of VAD, and the large number of children exposed to the procedure over the years, there is surprisingly limited information available regarding long–term outcomes in these children. Existing long–term follow–up studies have not revealed any significant differences in neurodevelopment outcome.[18-24]

The aim of the present study was to study neurodevelopment in children delivered at term by low- or mid-station station VAD compared to children born after ECD at the second stage of labor and to SVD. A validated screening method was used to screen for neurodevelopmental difficulties and registered neurodevelopmental ICD-10 diagnoses were collected.

Methods

Study design and study population

This was a prospective observational cohort study. We included all women delivered with a low– or mid-station station VAD at Karolinska University Hospital Huddinge, Sweden between 2007 and 2009 (n =493). Each individual extraction protocol was examined to confirm the classification of low– and mid-station station VADs according to international guidelines.[6, 17, 25] Exclusion criteria were outlet station VADs, preterm delivery <37+0 gestational weeks (n=19), BMI <18, and multiple pregnancies (n=6), with a final cohort of 468 children.

One comparison group consisted of women and their children delivered with SVD at Karolinska University Hospital Huddinge, the same date and time as the VAD group, but three years later, during 2010–2012 (n =870). After applying the exclusion criteria, the final group consisted of 869 children. In addition, we identified all cases of ECD at the second stage of labor with fetal heads at least at station 0 by scrutinizing the medical records. As this delivery mode was believed to be a rarer event, we included all women at both delivery wards at the Karolinska University Hospital, Huddinge and Solna, Stockholm, Sweden between 2007 and 2014 (n=184). Due to sample size, the range of age in these children was wider, six to 13 years of age instead of seven to ten, still suitable for the screening method.

The VAD followed national and international guidelines consisting of limiting the procedure to 20 minutes, a maximum of two cup–detachments and six pulls.[6, 17, 25, 26] The use of a Bird metal cup (50mm) and a vacuum pressure of 80kPa was standard practice. Both the VAD and the

Confidential: For Review Only

ECD procedures were exclusively carried out by physicians. SVDs were managed by a midwife, according to routine procedures in the Swedish Health Care system.
The annual delivery rate at the delivery ward Karolinska University Hospital Huddinge was 4500 during the study period, including vacuum extraction in 7–9 % of the deliveries. Fifty percent of all vacuum-assisted deliveries were outlet extractions during this period (station +5), and the frequency of failed extractions among all vacuum extractions was 7.1%.

Long-term follow-up
The Five–to Fifteen Questionnaire (FTF) was used as a primary long–term follow up of the children. It is a validated screening method for behavioral and neurodevelopmental difficulties developed in the Nordic countries in 2004.[27] It is aimed at parents, covering different aspects of the child´s functioning in daily life. The normative data is a stratified sample of 854 Swedish children from the general population. It is subdivided into three age groups (6–8 years, 9–12 years and 13–15 years) and presented for boys and girls of the Swedish norm group. There is a revised version of the FTF to be used between five and 17 years of age, validated in a Danish population reference, comparable to the original version.[28]
It comprises 181 items divided in to eight main domains (motor skills, executive functions, perception, memory, language, learning, social skills, and emotional/behavioral problems). The statements, answered by parents, are related to neurodevelopmental difficulties that can be endorsed as either: does not apply (score 0), applies sometimes or to some extent (score 1), or definitely applies (score 2). The 90th percentile is calculated and compared to normative data, used as a cut–off for obvious reported difficulties.
Study participants were contacted by mail including the FTF in Swedish. After two months, a reminding letter was sent out to the non–responding families and a final request was done by phone after another four weeks.
The parents chose if one or both were responsible for completing the questionnaire. A pediatric neurologist with no information on delivery mode reviewed the unidentified results by group ($</\geq$90th percentile) for each domain to account for non-random misclassification. The results were transferred to 5–15.org for calculation.

Data acquisition
Maternal, obstetric, and neonatal characteristics were collected from computerized delivery and neonatal charts (Obstetrix®) for the whole cohort.
Registered ICD–10 diagnoses for the participating children within each group of delivery mode were acquired from the national patient register at the Swedish National Board of Health and Welfare (SNBHW) until 2021. Included ICD–10 codes were F70–73, F78–79 (mental retardation), F80–84, F88.9 and F89.9 (disorders of the psychological development), F90–95, F98–99 (behavioral and emotional disorders), R48 (dyslexia and other symbolic dysfunctions), G80 (cerebral palsy), and G40 and G41 (episodic and paroxysmal disorders). The Swedish national patient register includes information on inpatient care since 1964 (full coverage since 1987) and outpatient specialist care since 2001. Main and secondary diagnoses were coded according to the ICD–system (tenth revision after 1998) by the assessing or discharging physician. The validity of most diagnoses, including psychiatric disorders, has proven to be high.[29]
Maternal educational level was collected from the longitudinal integration data base for health insurance and market studies (LISA), Statistics Sweden (Statistiska centralbyrån, SCB). LISA, includes yearly updated information on educational level on all individuals >16 years of age. The SUN2000 classification (Svensk UtbildningsNomenklatur, revised in 2000) divides education level into six categories: 0) preschool education; 1) pre– high school education <9 years; 2) pre– high school education 9–10 years; 3) high school education; 4) post– high school

Confidential: For Review Only

education <2 years; 5) post– high school education ≥2 years; and 6) doctoral studies/PhD. A subdivision of post– high school studies yes/no was done (levels 4–6).

Outcomes

The primary outcome was the FTF score for each main domain according to delivery mode. Secondary outcomes were the incidence of scoring ≥90th percentile in ≥2 main domains in the FTF for each delivery mode, and the incidence of an ICD–10 pediatric neurodevelopmental diagnosis in the study cohort according to the Swedish out–patient and in–patient registers.

Statistical analysis

IBM® SPSS® Statistics (version 27) was used as the statistical software and p-values of <0.05 were considered statistically significant. Descriptive statistics are presented as mean ± standard deviation (SD), median (min–max), or numbers and percentages as appropriate. The Chi square test was used for dichotomous data. Independent samples Student t-test or Wilcoxon rank sum tests were used for continuous variables. Univariate regression analyses were performed comparing: 1) VAD vs. SVD; 2) ECD vs. SVD and 3) VAD vs. ECD, with the last one serving as the reference group for each main domain.

To assist in choosing the variables in the multivariable regression analyses, a Directed Acyclic Graph (DAG) was performed, investigating potential confounders in the relationship between delivery mode and outcome (response in the FTF). The variables chosen for inclusion were maternal height (in centimeters), parity, maternal educational level, gestational length (in days), the use of oxytocin, and the child's age when the FTF was filled out. A multivariate regression analysis with the same comparisons as those in the univariate regression analysis was performed. When comparing VAD vs. ECD in the multivariate regression analysis, the variable 'indication for delivery ominous fetal heart rate' (OFHR) was included instead of oxytocin.

Public and Patient Involvement statement

There was no public or patient involvement in this study.

Results

1455 families were invited to participate (Figure S1). The response rate was 58% in the VAD group (n =253), 29% in the SVD group (n =247) and 50% in the ECD group (n =86).

A descriptive analysis on responders and non-responders in the VAD group is presented in Table 1. Except for more mid-station VADs in the non-response group, no other differences were found between the groups.

Table 1: Descriptive analysis of respondents and non-respondents in the vacuum-assisted delivery group

	FTF (n =253)	No FTF (n =187)	p-value
**Maternal characteristics**			
BMI (kg/m^2)[3]	23 (16-50)[§]	23 (17-39)[†]	0.82[6]
Nulliparous[1]	208 (82%)	146 (78%)	0.28[4]
Previous caesarean[1]	23 (9%)	20 (11%)	0.58[4]
Gestational length, days[2]	281±8	282±9	0.30[5]
**Delivery characteristics**			
First stage of labour, hours[2]	9±5	9±5	0.56[5]
Second stage of labour, hours[2]	2±2	2±2	0.22[5]

Confidential: For Review O

Epidural[1]	175 (72%)	135 (78%)	0.22[4]
Oxytocin[1]	242 (96%)	172 (92%)	0.11[4]
Indication OFHR[1]	108 (43%)	89 (48%)	0.31[4]
Station Mid[1]	148 (59%)	133 (71%)	<0.05[4*]
Number of pulls, n[3]	4 (1-10)	4 (1-13)	0.4[6]
Time during vacuum extraction, min[3]	7 (1-31)	7 (1-29)	0.94[6]
Position OAP[1]	221 (87%)	159 (85%)	0.48[4]
Subjectively heavy extraction[1]	58 (25%)	48 (28%)	0.44[4]
Failed vacuum extraction[1]	34 (13%)	33 (18%)	0.23[4]
Cup detachment[1]	39 (15%)	29 (16%)	0.98[4]
Shoulder dystocia[1]	11 (4%)	13 (7%)	0.23[4]
**Perinatal characteristics**			
Birth weight, g[2]	3668 ± 447	3641 ± 455	0.54[5]
Gender male[1]	138 (55%)	118 (63%)	0.07[4]
pH<7.00[1]	4 (2%)[‡]	0[□]	0.09[4]
Apgar<7 at 5 min[1]	9 (4%)	9 (5%)	0.51[4]
Severe outcome[1] (ICH, HIE, seizures, subgaleal haematoma)	4 (2%)	5 (3%)	0.42[4]

*Statistically significant at p-value <0.05. [1]n (%), [2]mean±sd, [3]median (min–max), [4]Chi[2] test, [5]Student T-test, [6]Wilcoxon rank sum test. [§]missing data 11%, [†]missing data 10%, [‡]missing data 18%, [□]missing data 19%. FTF: Five- to Fifteen questionnaire. BMI: body mass index, OFHR: ominous foetal heart rate, OAP: occipital-anterior position, ICH: intracranial haemorrhage, HIE: hypoxic ischemic encephalopathy

Confidential: For Review Only

A descriptive comparison was performed comparing the three delivery modes (Table 2). The analyses revealed several significant differences between the groups, mainly when comparing the instrumental delivery modes to the SVD group. There was a significantly higher proportion of nulliparous women, women with a previous cesarean section, and epidurals- and oxytocin usage in the VAD and ECD groups compared to the SVD group. There was a small difference between the educational level in the three groups, with a lower percentage of women with post-high school education in the VAD group. The duration of the second stage was longer in the VAD and ECD groups, and significantly longer in the ECD group compared to the VAD group. Compared to OFHR, dystocia was the most frequent indication for assisted delivery, most often with an ECD. Likewise, admission to NICU was more common after VAD.

Table 2: Descriptive analysis according to delivery mode

	VAD (n =253)	SVD (n =247)	ECD (n =86)	p-value
**Maternal characteristics**				
Nulliparous[1,4]	208 (82%)	151 (61%)	67 (78%)	<0.001[a*]<br><0.01[b*]<br>0.38[c]
Previous caesarean[1,4]	23 (9%)	4 (2%)	11 (13%)	<0.001[a*]<br><0.001[b*]<br>0.32[c]
Post- high school education[1,4]	166 (66%)	185 (75%)	61 (71%)	<0.05[a*]<br>0.5[b]<br>0.4[c]
BMI (kg/m[2])[3,6]	23 (18-50)	23 (18-35)	23 (18-37)	0.42[a]<br><0.01[b*]<br><0.05[c*]
Gestational length, days[3,6]	281 (261-295)	279 (259-294)	281 (259-295)	<0.001[a*]<br><0.001[b*]

				<0.05[c*]
First stage of labour, hours[2,5]	8.8 (±4.6)	4.2 (±3.7)	8.4 (±5.2)	<0.001[a*]<br><0.001[b*]<br>0.53[c]
Second stage of labour, hours[3,6]	2 (0-6.5)	0.5 (0-5)	5 (0-17)	<0.001[a*]<br><0.001[b*]<br><0.001[c*]
**Delivery characteristics**				
Indication OFHR	108 (43%)		22 (26%)	<0.01[c*]
Indication dystocia	145 (57%)		60 (74%)	
Epidural[1,4]	183 (72%)	78 (32%)	69 (80%)	<0.001[a*]<br><0.001[b*]<br>0.2[c]
Oxytocin[1,4]	242 (96%)	99 (40%)	83 (97%)	<0.001[a*]<br><0.001[b*]<br>0.7[c]
Position OAP[1,4]	221 (90%)	237 (96%)	61 (71%)	<0.05[a*]<br><0.001[b*]<br><0.001[c*]
Shoulder dystocia[1,4]	11	0		0.001[a*]
**Perinatal characteristics**				
Birth weight, g[2,5]	3668 (±447)	3578 (±429)	3836 (±494)	<0.05[a*]<br><0.001[b*]<br><0.01[c*]
Gender male[1,4]	115 (46%)	119 (48%)	36 (42%)	0.5[a]<br>0.3[b]<br>0.6[c]
NICU admission[1,4]	40 (16%)	1 (0.4%)	5 (6%)	<0.001[a*]<br>0.001[b*]<br><0.05[c*]
pH a. umbilicalis<7.00[1,4]	4	0	0	<0.05[a*]<br>0.2[c]
Apgar<7 at 5 min[1,4]	9	0	0	<0.01[a*]<br>0.08[c]

*Statistically significant at p-value <0.05. *a: VAD vs. SVD, b: ECD vs. SVD, c: VAD vs. ECD.* [1]n (%), [2]mean± sd, [3]median (min-max), [4]Chi^2 test, [5]Student T-test, [6]Wilcoxon rank sum test. BMI: body mass index, OFHR: ominous foetal heart rate, OAP: occipital-anterior position, NICU: neonatal intensive care unit

Confidential: For Review Only

The Five-to-Fifteen questionnaire

A basic characteristics comparison between children with obvious difficulties in one or more domains according to the answers in the FTF (≥90th percentile) and children without difficulties (<90th percentile), according to normative data, was performed for each delivery mode. For all delivery modes, there were statistically significantly more women with a post-high school education in the group with children scoring <90th percentile. Apart from this, there were more subjectively heavy extractions among children scoring ≥90th percentile born after VAD (p-value <0.05). In the ECD group there were more males among children scoring <90th percentile (p-value <0.01). The mean age of the children when the FTF was completed was eight (±0.9) in the VAD group, eight (±0.9) in the SVD group and nine (±2.2) in the ECD group. When comparing the VAD and the SVD group to the ECD group, this age difference was statistically significant (p<0.001 in both comparisons).

Three univariate regression analyses are presented in Table 3 (VAD vs. SVD, ECD vs. SVD and VAD vs. ECD). There was a significantly higher proportion of children scoring to have

problems linked to motor skills and perception in the VAD group compared to the SVD group. The same results were seen when comparing ECD to SVD. There were no calculated differences between the VAD and ECD groups.

Table 3: Number and percentages of children scoring ≥90th percentile in the FTF per main domain; a univariate logistic regression analysis

Main domain	VAD n/total (%)	SVD n/total (%)	ECD n/total (%)	OR (95% CI)	p-value
Motor skills	47/253 (19%)	23/246 (9%)	22/86 (26%)	2.2 (1.3- 3.8)[a]	<0.01[a]*
				3.3 (1.8-6.4)[b]	<0.001[b]*
				0.7 (0.4-1.2)[c]	0.2[c]
Executive functions	40/253 (16%)	34/247 (14%)	10/86 (12%)	1.2 (0.7-1.9)[a]	0.5[a]
				0.8 (0.4-1.8)[b]	0.6[b]
				0.8 (0.4-1.8)[c]	0.6[c]
Perception	42/253 (17%)	26/247 (11%)	18/86 (21%)	1.7 (1.002-2.9)[a]	<0.05[a]*
				2.3 (1.2-4.4)[b]	<0.05[b]*
				0.8 (0.4-1.4)[c]	0.4[c]
Memory	29/253 (11%)	26/247 (11%)	5/86 (6%)	1.1 (0.6-1.9)[a]	0.7[a]
				0.5 (0.2-1.4)[b]	0.2[b]
				2.1 (0.8-5.6)[c]	0.1[c]
Language	31/253 (12%)	22/247 (9%)	6/86 (7%)	1.4 (0.8-2.5)[a]	0.2[a]
				0.8 (0.3-2)[b]	0.9[b]
				1.9 (0.8-4.6)[c]	0.2[c]
Learning	30/195 (15%)	23/192 (12%)	7/62 (11%)	1.3 (0.8-2.4)[a]	0.3[a]
				0.9 (0.4-2.3)[b]	0.9[b]
				1.4 (0.6-3.4)[c]	0.4[c]
Social skills	30/252 (12%)	19/246 (8%)	6/81 (7%)	1.6 (0.9-3)[a]	0.1[a]
				0.9 (0.4-2.5)[b]	0.9[b]
				1.7 (0.7-4.2)[c]	0.3[c]
Emotional/ Behavioural problems	45/253 (18%)	29/247 (12%)	13/86 (15%)	1.6 (0.9-2.7)[a]	0.06[a]
				1.3 (0.7-2.7)[b]	0.4[b]
				1.2 (0.6-2.4)[c]	0.6[c]

*Statistically significant at p-value <0.05. *a: VAD vs. SVD, b: ECD vs. SVD, c: VAD vs. ECD.* FTF: Five-to-Fifteen questionnaire. VAD: vacuum-assisted delivery, SVD: spontaneous vaginal delivery, ECD: emergency caesarean delivery. OR: odds ratio

Confidential: For Review Only

Table 4 presents the number and percentage of children scoring ≥90th percentile in two or more of the main domains. There were significantly more children with obvious difficulties in two or more domains in the VAD and ECD groups compared to the SVD group.

Table 4: Number and percentages of children scoring ≥90th percentile in the FTF in ≥2 domains; a univariate logistic regression analysis with OR and 95% CI

	VAD n/total (%)	SVD n/total (%)	ECD n/total (%)	OR (95%CI)	p-value
≥2 domains	65/87 (75%)	37/73 (49%)	22/31 (71%)	1.96 (1.3-3.1)[a]<br>1.95 (1.1-3.6)[b]<br>1.00 (0.6-1.8)[c]	<0.01[a*]<br><0.05[b*]<br>0.98[c]

*Statistically significant at p-value<0.05. *a: VAD vs. SVD b: ECD vs. SVD c: VAD vs. ECD.* FTF: Five-to-Fifteen questionnaire, VAD: vacuum-assisted, SVD: spontaneous vaginal delivery, ECD: emergency caesarean delivery

A multivariable regression analysis was performed using the exposure delivery mode and the FTF outcome according to each main domain. The potential confounders included are described in the method section. Children delivered with a VAD had significantly more problems with motor skills than children delivered with a SVD (aOR 2.5, 95% CI: 1.2–5.3). The same was seen when comparing ECD to SVD (aOR 3.3, 95% CI: 1.4–7.9). In the comparison between VAD and ECD, the variable indication OFHR was included instead of oxytocin infusion. No differences were seen between the groups.

The diagnoses acquired from the SNBHW are presented in Table 5. There are significantly more children with attention deficit/hyperactivity disorder in the VAD group compared to the other two delivery modes. This is also true for autism spectrum disorders when comparing VAD to ECD. In general, children delivered by VAD had significantly more often one diagnosis, or more, than children delivered by SVD (p <0.001). This difference was not seen when comparing VAD to ECD (p =0.08).

Table 5: Children´s diagnoses according to ICD-10

	VAD (n =253)	SVD (n =247)	ECD (n =86)	p-value
Communication disorders (F80)	5 (2%)	1 (0.4%)	0	0.2[a]<br>1[b]<br>0.3[c]
Specific developmental disorder of motor function (F829)	2 (0.8%)	0	0	0.5[a]<br>-<br>1[c]
Autism spectrum disorders (F84)	12 (5%)	6 (2%)	0	0.2[a]<br>0.3[b]<br><0.05[c*]
Unspecified disorder of psychological development (F899)	0	0	1 (1%)	-<br>0.3[b]<br>0.3[c]
Attention deficit hyperactivity disorder (F90)	31 (12%)	8 (3%)	0	<0.001[a*]<br>0.1[b]<br><0.001[c*]
Oppositional defiant disorder (F913)	1 (0.4%)	0	0	1[a]<br>-<br>1[c]
Tic disorders (F95)	4 (2%)	0	0	0.1[a]<br>-<br>0.6[c]
Enuresis (F980)	4 (2%9	4 (2%)	1 (1%)	0.9[a]<br>0.8[b]<br>0.8[c]
Encopresis (F981)	1 (0.4%)	0	3 (3%)	1[a]

Confidential: For Review Only

				<0.05[b*]<br>0.9[c]
Developmental and emotional disorders (F98)	2 (0.8%)	0	1 (1%)	0.2[a]<br>0.09[b]<br>0.8[c]
Unspecified mental disorder (F999)	3 (1%)	2 (0.8%)	0	0.7[a]<br>0.4[b]<br>0.3[c]
Epilepsy (G40)	2 (0.8%)	1 (0.4%)	2 (2%)	0.6[a]<br>0.1[b]<br>0.3[c]
Neurodevelopmental diagnosis	37 (15%)	14 (6%)	7 (8%)	0.001[a*]<br>0.5[b]<br>0.1[c]
Child with ≥2 neurodevelopmental diagnoses	20 (8%)	6 (2%)	2 (2%)	<0.001[a*]<br>1[b]<br>0.08[c]

*Significant at p-value <0.05 calculated with the Chi² test. N (%). *A: VAD vs. SVD, b: ECD vs. SVD, c: VAD vs. ECD.* VAD: vacuum-assisted delivery, SVD: spontaneous vaginal delivery, ECD: emergency caesarean delivery

Discussion

Main findings

According to our findings, low- and mid-station VADs imply increased odds of long-term neurodevelopmental difficulties in motor skills and perception between seven and ten years of age compared to spontaneously delivered children. Similar findings were seen in the group delivered with an ECD. There were no calculated differences in outcome when comparing VAD to ECD. After adjusting for the proposed confounding variables, the multivariable regression analysis showed that the increased odds for difficulties in motor skills after a VAD or an ECD was maintained.

Considering the relatively frequent use of VAD in modern obstetrics, it is striking how little is known about the long-term outcomes. According to the current study, this group of children have an increased risk of experiencing neurodevelopmental difficulties such as motor skills and perception. On the other hand, it cannot be concluded that VAD itself causes these results, since children born after an ECD also exhibit a similarly risk. The adjustment for the confounding variable 'indication' controls for confounding by indication in the relationship between the exposure and the outcome in the comparison between VAD and ECD. The fact that no significant difference was seen in this comparison suggest that rather than the intervention itself, it appears that the complete situation, including the lack of progress in the second stage of labor, and the maternal and fetal conditions, may be responsible for the problematic outcome. It appears, however, that children born after VAD might be at an elevated risk of ADHD, as indicated by the register data. This increased incidence was not observed in the ECD group, but due to the limited sample size in the ECD group, caution is needed when evaluating these results.

Strengths and limitations

To our knowledge, this is the first study on a well-defined cohort of children delivered by low- and mid-station VADs investigating long-term outcomes using a validated screening method. Including the registered neurodevelopmental ICD-10 diagnoses at the SNBHW, the value of the study is enhanced.

Confidential: For Review Only

Despite the fact that the validated questionnaire already contains a normative reference group, we decided to include a control group with children born via SVD from the same delivery unit and region to increase validity. In addition, the included group of children born after ECD at the second stage of labor with a fetal head station at level 0 or more, without a prior VAD attempt, was believed to account for confounding by indication and to make the groups more comparable. This group had a significantly longer duration of the second stage (a median of five hours) and showed similar results in the FTF as the VAD group. On the other hand, there were more children with neurodevelopmental diagnoses and NICU admissions in the VAD group.

The FTF questionnaire is a validated method for neurodevelopmental screening in children five years of age and up.[27, 28, 30-32] It might be questioned whether parents' estimates of a child's development are objective. However, the FTF is not a diagnostic tool, and a multi-professional assessment is needed to diagnose neurodevelopmental disorders in children and youth. Since the questionnaire was distributed only in the Swedish language, an inevitable selection of parents might exist. This could be seen as a limitation, affecting the results, but the same selection was made in the three study groups, and thus any resulting limitation applies to the whole cohort.

The low response rate in the SVD group (29%) is a limitation and could be attributed to the fact that a control group is less likely to respond. The response analysis in the VAD group did not reveal any differences, except a higher rate of mid-station vacuum extraction in the non-respondent group.

Interpretation

The study is observationally designed to screen for neurodevelopmental difficulties rather than to reveal any causal relationships. As time interval between exposure (delivery) and outcome is a matter of years during a child´s development, a direct cause cannot be proven. However, with the use of the FTF we were able to study several neurodevelopmental aspects, presented as the main domains in Table 3. The results from the present study are in contrast with the few existing ones reporting on specific long–term neurodevelopmental aspects[18-20, 22, 33], but these have not stratified according to the VAD type, they have smaller cohorts, and did not use validated follow–up methods.

Regarding the results related to the children's diagnoses, it is interesting to note that even though many have motor skill problems according to the FTF, they do not have individual motor skill diagnoses. These are rather revealed in a more complex form i.e., as a comorbidity in an ADHD, which could be due to an underdiagnosis of motor skill problems. If the child had the diagnosis when the questionnaire was filled out or if it was diagnosed later on is not known, as time between the FTF completion and register extract was almost two years.

Whether or not low– and mid-station station VADs or ECD at the second stage of labor affects specific parts of the fetal brain resulting in impaired neurodevelopment for different skills, is unknown. Although the etiology of diagnoses such as ADHD is heterogeneous and unclear, it is known to be associated with traumatic brain injury.[34] Apart from this, there is an established association between ADHD and heredity.[35, 36] Unfortunately, due to the lack of an ethical approval, registered neurodevelopmental diagnoses among parents or siblings were not collected.

We included the maternal educational level as a proxy for socioeconomic status, since most questionnaires were filled out by mothers. According to the descriptive analyses for the different delivery modes, mothers with higher educational levels more often had children scoring <90th percentile, and mothers in the VAD group had significantly lower educational

Confidential: For Review Only

level than the other two groups. This variable was adjusted for in the multivariate regression analysis.

Conclusion

Obstructed labor at the second stage in term deliveries is a delicate mission for clinicians. Although interpreted with caution, the significant difference in long-term neurodevelopmental difficulties in children delivered with a VAD and ECD compared to a SVD is worrying. These results demand further studies, preferably in prospective designs, including both VAD and ECD at the second stage, using structured long-term follow-ups. If additional studies show similar results, it seems necessary to revise the existing guidelines for VAD and ECD at the second stage.

Ethical approval Ethical approval was obtained by the Regional Ethical Review Board Stockholm, Sweden (DNR 2014/1860–31, DNR 2017/1411–32, DNR 2019/04880 and DNR 2021/02457).

Funding statement This work was supported by the Stockholm City Council (Agreement for Research and Education of Doctors) (grant number FoUI-953029 and FoUI-955070), CIMED (Center for Innovative Medicine) (grant number FoUI-954462), Karolinska Institutet and General Maternity Hospital Foundation (Stiftelsen Allmänna BB:s Minnesfond) (grant number N/A).

Competing interest statement All authors have completed the ICMJE uniform disclosure form at http://www.icmje.org/disclosure-of-interest/ and declare: no support from any organisation for the submitted work; no financial relationships with any organisations that might have an interest in the submitted work in the previous three years; no other relationships or activities that could appear to have influenced the submitted work.

Authors' contributions GA and MW designed the study, acquired, analysed, interpreted the data, and wrote and revised the manuscript. SR acquired, analysed, interpreted the data, and wrote and revised the manuscript. KL analysed, interpreted the data, and revised the manuscript. The corresponding author attests that all listed authors meet authorship criteria and that no others meeting the criteria have been omitted.

STROBE checklist see supplementary files

Data sharing statement "The Corresponding Author has the right to grant on behalf of all authors and does grant on behalf of all authors, if the manuscript is accepted, a worldwide licence to the Publishers and its licensees in perpetuity, in all forms, formats and media (whether known now or created in the future), to i) publish, reproduce, distribute, display and store the Contribution, ii) translate the Contribution into other languages, create adaptations, reprints, include within collections and create summaries, extracts and/or, abstracts of the Contribution, iii) create any other derivative work(s) based on the Contribution, iv) to exploit all subsidiary rights in the Contribution, v) the inclusion of electronic links from the Contribution to third party material wherever it may be located; and, vi) licence any third party to do any or all of the above."

Transparency declaration: The lead author (GA) affirms that the manuscript is an honest, accurate, and transparent account of the study being reported; that no important aspects of the study have been omitted and that any discrepancies from the study as planned have been explained.

Summary box

What is already known on this topic

Confidential: For Review Only

- There are very few studies investigating the long–term neurodevelopmental difficulties in children born after a vacuum-assisted delivery, and there is an evident lack of information.
- No differences were reported in previous studies on long-term neurodevelopmental outcomes after vacuum-assisted delivery.
- Several methodological issues have been identified in previous studies, including limited information regarding the vacuum-assisted procedure and the use of non-validated methods for neurodevelopmental screening.

What this study adds

- Children born after low– or mid-station station vacuum-assisted deliveries may be at an increased risk for long–term neurodevelopmental difficulties in comparison to those born after a spontaneous vaginal delivery.
- Similar results were seen in the group of children born after an emergency caesarean delivery at the second stage of labour compared to those born after a spontaneous vaginal delivery.
- Children delivered with a vacuum-assisted delivery may have more attention deficit/hyperactivity disorders than children born after the other two delivery modes.

How this study might affect research, practice or policy

- Considering the frequent use of the VAD and the ECD in the second stage of labour, the unexpected results of an increased odds of long-term problems in motor skills and perception in these children is striking and worrying.
- These results need to be promptly investigated, preferably in prospective studies. If similar results are seen, the clinical guidelines for these two methods need to be revised.

Confidential: For Review Only

References

1. Malmstrom T. Vacuum extractor, an obstetrical instrument. *Acta Obstet Gynecol Scand Suppl*. 1954;33(4):1-31.
2. Martin Ja Fau - Hamilton BE, Hamilton Be Fau - Osterman MJK, Osterman Mjk Fau - Driscoll AK, Driscoll Ak Fau - Drake P, Drake P. Births: Final Data for 2017. (1551-8922 (Print))
3. The Swedish National Board of Health and Welfare T. Statistikbas för graviditeter, förlossningar och nyfödda. https://www.socialstyrelsen.se/statistik-och-data/statistik/alla-statistikamnen/graviditeter-forlossningar-och-nyfodda/
4. NHS. NHS Maternity Statistics, England. https://digital.nhs.uk/data-and-information/publications/statistical/nhs-maternity-statistics/2020-21
5. Canadian Institute for Health Information. Assisted delivery rate (overall) among vaginal deliveries. https://apps.cihi.ca/mstrapp/asp/Main.aspx?evt=2048001&documentID=029DB170438205AEBCC75B8673CCE822&Server=apmstrextprd_i&Project=Quick+Stats&
6. (RANZCOG) TRNZCoOaG. *Instrumental vaginal birth*. 2020. https://ranzcog.edu.au/RANZCOG_SITE/media/RANZCOG-MEDIA/Women%27s%20Health/Statement%20and%20guidelines/Clinical-Obstetrics/Instrumental-vaginal-birth-(C-Obs-16)-Review-March-2020.pdf?ext=.pdf
7. Macfarlane AJ, Blondel B, Mohangoo AD, et al. Wide differences in mode of delivery within Europe: risk-stratified analyses of aggregated routine data from the Euro-Peristat study. *Bjog*. Mar 2016;123(4):559-68. doi:10.1111/1471-0528.13284
8. Ekeus C, Hogberg U, Norman M. Vacuum assisted birth and risk for cerebral complications in term newborn infants: a population-based cohort study. *BMC Pregnancy Childbirth*. Jan 20 2014;14:36. doi:10.1186/1471-2393-14-36
9. Muraca GM, Boutin A, Razaz N, et al. Maternal and neonatal trauma following operative vaginal delivery. *CMAJ*. Jan 10 2022;194(1):E1-E12. doi:10.1503/cmaj.210841
10. Gebremariam A. Subgaleal haemorrhage: risk factors and neurological and developmental outcome in survivors. *Ann Trop Paediatr*. Mar 1999;19(1):45-50. doi:10.1080/02724939992626
11. Towner DR, Ciotti MC. Operative vaginal delivery: a cause of birth injury or is it? *Clin Obstet Gynecol*. Sep 2007;50(3):563-81. doi:10.1097/GRF.0b013e31811eaa39
12. Walsh CA, Robson M, McAuliffe FM. Mode of delivery at term and adverse neonatal outcomes. *Obstet Gynecol*. Jan 2013;121(1):122-8. doi:http://10.1097/AOG.0b013e3182749ac9
10.1097/aog.0b013e3182749ac9
13. Halscott TL, Reddy UM, Landy HJ, et al. Maternal and Neonatal Outcomes by Attempted Mode of Operative Delivery From a Low Station in the Second Stage of Labor. *Obstet Gynecol*. Dec 2015;126(6):1265-72. doi:10.1097/AOG.0000000000001156

Confidential: For Review Only

14. Muraca GM, Skoll A, Lisonkova S, et al. Perinatal and maternal morbidity and mortality among term singletons following midcavity operative vaginal delivery versus caesarean delivery. *BJOG*. May 2018;125(6):693-702. doi:10.1111/1471-0528.14820

15. Muraca GM, Sabr Y, Lisonkova S, et al. Perinatal and maternal morbidity and mortality after attempted operative vaginal delivery at midpelvic station. *CMAJ*. Jun 5 2017;189(22):E764-E772. doi:10.1503/cmaj.161156

16. Vacca A. Reducing the Risks of a Vacuum Delivery. *Fetal Medicine Review*. 2006;17 (4):301-315. doi:doi:10.1017/S0965539506001823

17. Murphy DJ, Strachan BK, Bahl R, Royal College of O, Gynaecologists. Assisted Vaginal Birth: Green-top Guideline No. 26. *BJOG*. Aug 2020;127(9):e70-e112. doi:10.1111/1471-0528.16092

18. Ahlberg M, Ekeus C, Hjern A. Birth by vacuum extraction delivery and school performance at 16 years of age. *Am J Obstet Gynecol*. Apr 2014;210(4):361 e1-361 e8. doi:10.1016/j.ajog.2013.11.015

19. Ngan HY, Miu P, Ko L, Ma HK. Long-term neurological sequelae following vacuum extractor delivery. *Aust N Z J Obstet Gynaecol*. May 1990;30(2):111-4.

20. Johanson RB, Heycock E, Carter J, Sultan AH, Walklate K, Jones PW. Maternal and child health after assisted vaginal delivery: five-year follow up of a randomised controlled study comparing forceps and ventouse. *Br J Obstet Gynaecol*. Jun 1999;106(6):544-9.

21. Bjerre I, Dahlin K. The long-term development of children delivered by vacuum extraction. *Dev Med Child Neurol*. Jun 1974;16(3):378-81. doi:10.1111/j.1469-8749.1974.tb03354.x

22. Seidman DS, Laor A, Gale R, Stevenson DK, Mashiach S, Danon YL. Long-term effects of vacuum and forceps deliveries. *Lancet*. Jun 29 1991;337(8757):1583-5.

23. Mastrolia SA, Wainstock T, Sheiner E, Landau D, Sergienko R, Walfisch A. Failed Vacuum and the Long-Term Neurological Impact on the Offspring. *Am J Perinatol*. Nov 2017;34(13):1306-1311. doi:10.1055/s-0037-1603507

24. Ayala NK, Schlichting LE, Kole MB, et al. Operative vaginal delivery and third grade educational outcomes. *Am J Obstet Gynecol MFM*. Nov 2020;2(4):100221. doi:10.1016/j.ajogmf.2020.100221

25. ACOG. Operative Vaginal Birth: ACOG Practice Bulletin, Number 219. *Obstet Gynecol*. Apr 2020;135(4):e149-e159. doi:10.1097/AOG.0000000000003764

26. Vladic-Stjernholm YWAA-WI. Instrumental delivery by vacuum extraction: advice for clinical recommendations (in Swedish): Löf. https://lof.se/filer/Instrumentell-forlossning-med-sugklocka.pdf

27. Kadesjo B, Janols LO, Korkman M, et al. The FTF (Five to Fifteen): the development of a parent questionnaire for the assessment of ADHD and comorbid conditions. *Eur Child Adolesc Psychiatry*. 2004;13 Suppl 3:3-13. doi:10.1007/s00787-004-3002-2

28. Lambek R, Trillingsgaard A. Elaboration, validation and standardization of the five to fifteen (FTF) questionnaire in a Danish population sample. *Res Dev Disabil*. Mar 2015;38:161-70. doi:10.1016/j.ridd.2014.12.018

29. Ludvigsson JF, Andersson E, Ekbom A, et al. External review and validation of the Swedish national inpatient register. *BMC Public Health*. Jun 9 2011;11:450. doi:10.1186/1471-2458-11-450

Confidential: For Review Only

30. Trillingsgaard A, Damm D, Sommer S, et al. Developmental profiles on the basis of the FTF (Five to Fifteen) questionnaire-clinical validity and utility of the FTF in a child psychiatric sample. *Eur Child Adolesc Psychiatry*. 2004;13 Suppl 3:39-63. doi:10.1007/s00787-004-3006-y
31. Korkman M, Jaakkola M, Ahlroth A, Pesonen AE, Turunen MM. Screening of developmental disorders in five-year-olds using the FTF (Five to Fifteen) questionnaire: a validation study. *Eur Child Adolesc Psychiatry*. 2004;13 Suppl 3:31-8. doi:10.1007/s00787-004-3005-z
32. Bohlin G, Janols LO. Behavioural problems and psychiatric symptoms in 5-13 year-old Swedish children-a comparison of parent ratings on the FTF (Five to Fifteen) with the ratings on CBCL (Child Behavior Checklist). *Eur Child Adolesc Psychiatry*. 2004;13 Suppl 3:14-22. doi:10.1007/s00787-004-3003-1
33. Nilsen ST. Boys born by forceps and vacuum extraction examined at 18 years of age. *Acta Obstet Gynecol Scand*. 1984;63(6):549-54. doi:10.3109/00016348409156718
34. Asarnow RF, Newman N, Weiss RE, Su E. Association of Attention-Deficit/Hyperactivity Disorder Diagnoses With Pediatric Traumatic Brain Injury: A Meta-analysis. *JAMA Pediatr*. Oct 1 2021;175(10):1009-1016. doi:10.1001/jamapediatrics.2021.2033
35. Larsson H, Chang Z, D'Onofrio BM, Lichtenstein P. The heritability of clinically diagnosed attention deficit hyperactivity disorder across the lifespan. *Psychol Med*. Jul 2014;44(10):2223-9. doi:10.1017/S0033291713002493
36. Faraone SV, Larsson H. Genetics of attention deficit hyperactivity disorder. *Mol Psychiatry*. Apr 2019;24(4):562-575. doi:10.1038/s41380-018-0070-0

Confidential: For Review Only

BMJ Paediatrics Open

Long-term neurodevelopmental outcome in children born after vacuum-assisted delivery compared to second stage caesarean delivery and spontaneous vaginal delivery - a cohort study

Journal:	*BMJ Paediatrics Open*
Manuscript ID	bmjpo-2023-002048.R1
Article Type:	Original research
Date Submitted by the Author:	08-Aug-2023
Complete List of Authors:	Romero, Stefhanie; Karolinska Institute Department of Clinical Science Intervention and Technology, Clinical science, intervention and technology; Karolinska University Hospital, Pregnancy Care and Delivery<br>Lindström, Katarina; Karolinska Institute Department of Clinical Science Intervention and Technology, Clinical Science Intervention and Technology; Karolinska University Hospital, Neuropaediatrics<br>Listermar, Johanna; Karolinska Institute<br>Westgren, Magnus; Karolinska Institute Department of Clinical Science Intervention and Technology, Clinical Science Intervention and Technology<br>Ajne, Gunilla; Karolinska Institute Department of Clinical Science Intervention and Technology, Clinical Science Intervention and Technology; Karolinska University Hospital, Pregnancy Care and Delivery
Keywords:	Psychology, Pathology, Neonatology, Epidemiology, Adolescent Health

SCHOLARONE™
Manuscripts

I, the Submitting Author has the right to grant and does grant on behalf of all authors of the Work (as defined in the below author licence), an exclusive licence and/or a non-exclusive licence for contributions from authors who are: i) UK Crown employees; ii) where BMJ has agreed a CC-BY licence shall apply, and/or iii) in accordance with the terms applicable for US Federal Government officers or employees acting as part of their official duties; on a worldwide, perpetual, irrevocable, royalty-free basis to BMJ Publishing Group Ltd ("BMJ") its licensees and where the relevant Journal is co-owned by BMJ to the co-owners of the Journal, to publish the Work in this journal and any other BMJ products and to exploit all rights, as set out in our licence.

The Submitting Author accepts and understands that any supply made under these terms is made by BMJ to the Submitting Author unless you are acting as an employee on behalf of your employer or a postgraduate student of an affiliated institution which is paying any applicable article publishing charge ("APC") for Open Access articles. Where the Submitting Author wishes to make the Work available on an Open Access basis (and intends to pay the relevant APC), the terms of reuse of such Open Access shall be governed by a Creative Commons licence – details of these licences and which Creative Commons licence will apply to this Work are set out in our licence referred to above.

Other than as permitted in any relevant BMJ Author's Self Archiving Policies, I confirm this Work has not been accepted for publication elsewhere, is not being considered for publication elsewhere and does not duplicate material already published. I confirm all authors consent to publication of this Work and authorise the granting of this licence.

Confidential: For Review Only

Long-term neurodevelopmental outcome in children born after vacuum-assisted delivery compared to second stage caesarean delivery and spontaneous vaginal delivery: a cohort study

Stefhanie Romero[1,2], MD PhD, Katarina Lindström[3,4], MD PhD, Johanna Listermar, Medical student[5], Magnus Westgren[1], MD PhD, Gunilla Ajne[1,2], MD PhD

[1]Division of Obstetrics and Gynaecology, CLINTEC, Karolinska Institutet, Stockholm, 171 77, Sweden
[2] Pregnancy Care & Delivery, Karolinska University Hospital, Stockholm, 141 86, Sweden,
[3]Division of Paediatrics, CLINTEC, Karolinska Institutet, Stockholm, 171 77, Sweden
[4]Neuropediatrics, Karolinska University Hospital, Stockholm, 141 86, Sweden
[5]Karolinska Institutet, Stockholm, 171 77, Sweden
Correspondence to: Stefhanie Romero, Pregnancy Care & Delivery, B76, Karolinska University Hospital, Stockholm, 141 86, Sweden. stefhanie.romero@ki.se.

Key message: When screening for long-term neurodevelopmental problems, children delivered with a low- or mid-station vacuum-assisted delivery or an emergency caesarean delivery at the second stage of labour were found to have significant difficulties with motor skills and perception compared to children delivered spontaneously.

Confidential: For Review Only

Abstract

Objective To evaluate long-term neurodevelopment in children born after low-or mid-station vacuum-assisted delivery (VAD) compared to children delivered by second stage cesarean delivery (SSCD) or spontaneous vaginal delivery (SVD).

Design Cross sectional cohort study

Setting Two delivery wards, Karolinska University Hospital, Sweden

Patients 253 children born by low– or mid–station VAD, 247 children born after an SVD, and 86 children born via an SSCD accepted to participate.

Interventions The Five-to-Fifteen questionnaire was used as a validated screening method for neurodevelopmental difficulties, assessed by parents.

Main outcomes measures Results in the Five-to-Fifteen questionnaire. In addition, registered neurodevelopmental ICD-10 diagnoses were collected. Regression analyses estimated associations between delivery modes.

Results Children born after VAD exhibited an increased rate of long–term neurodevelopmental difficulties in motor skills (OR 2.2, 95%CI 1.3 to 3.8) and perception (OR 1.7, 95%CI 1.002 to 2.9) compared to SVD. Similar findings were seen in the group delivered with an SSCD compared to SVD (motor skills: OR 3.3, 95%CI 1.8 to 6.4 and perception: OR 2.3, 95%CI 1.2 to 4.4). The increased odds for motor skills difficulties after VAD and SSCD remained after

adjusting for proposed confounding variables. There were significantly more children in the VAD group with registered neurodevelopmental ICD–10 diagnoses such as attention deficit/hyperactivity disorders.

Conclusions The differences in long–term neurodevelopmental difficulties in children delivered with a VAD or SSCD compared to SVD in the present study, indicate the need for increased knowledge in the field to optimize the management of second stage of labor.

Confidential: For Review Only

Introduction

The vacuum extractor is used in cases of dystocia or foetal distress during the second stage of labour.[1] The incidence of vacuum-assisted delivery (VAD) varies worldwide, representing up to 3% among all vaginal births in the USA[2], and 5–10% in Europe, Canada, and Australia/New Zealand.[3-7]
Serious foetal complications are rare after VAD, but appears to have a higher rate of intracranial haemorrhage (0.1-0.8%)[8] and subgaleal hematomas (0.4 - 2.5%)[9] compared to spontaneous vaginal delivery (SVD).[10 11] Results are not consistent when comparing VAD to other instrumental delivery modes, including caesarean delivery at the second stage of labour (SSCD).[9 12 13] Some studies indicate an increased risk of birth trauma in the VAD group[14], while others report no differences in severe neonatal outcomes.[15 16] The most appropriate comparison to VAD is SSCD since both are optional delivery methods to a SVD at this point.
VAD is classified according to foetal head station in the birth canal into mid, low and outlet stations, the last of which is considered a low risk VAD.[17 18] Previous research has not always taken this stratification into account, which could be one reasons behind the divergent results.

Considering the large number of children exposed to VAD, there is limited information regarding long–term outcomes. Existing studies have not revealed any significant differences in neurodevelopment outcome.[19-25]

The aim of the present study was to study neurodevelopment in children delivered at term by low- or mid-station station VAD compared to children born after SSCD and SVD.

Methods

Study design and study population

This was a cross sectional cohort study, including all women delivered with a low– or mid-station VAD at Karolinska University Hospital Huddinge, Sweden between 2007 and 2009 (n =493). Each individual extraction protocol was examined to confirm the classification of foetal station.[6 18 26] Exclusion criteria were outlet station VADs, preterm delivery <37+0 gestational weeks (n=19), BMI <18, and multiple pregnancies (n=6), with a final number of 468 children.

The SVD group consisted of women and their children delivered at Karolinska University Hospital Huddinge. This group was included three years after the VAD group but matched by calendar date and time of birth (2010-2012, n =870). After applying the exclusion criteria, the final group consisted of 869 children. In addition, we identified all cases of SSCD with foetal heads at least at station 0 by scrutinizing the medical records. As this delivery mode was expected to be a rarer event, we included all women at both delivery wards at the Karolinska University Hospital, Huddinge and Solna, between 2007 and 2014 (n=184).

The VAD followed national and international guidelines consisting of limiting the procedure to 20 minutes, a maximum of two cup–detachments and six pulls.[6 18 26 27] The use of a Bird metal cup (50mm) and a vacuum pressure of 80kPa was standard. Both the VAD and the SSCD procedures were exclusively carried out by physicians. SVDs were managed by a midwife, according to routine procedures in the Swedish Health Care system.
A predefined response analysis was included in the study protocol for the VAD group.

Long-term follow-up
The Five–to Fifteen Questionnaire (FTF) was used as a primary long–term follow up. It is a validated screening method for behavioural and neurodevelopmental difficulties.[28] It is aimed at parents, covering different aspects of the child´s functioning. It is subdivided into three age groups (6–8 years, 9–12 years and 13–15 years) and presented for boys and girls of a Swedish norm group.[28-31]
It comprises 181 items divided into eight main domains (motor skills, executive functions, perception, memory, language, learning, social skills, and emotional/behavioural problems). The 90th percentile is calculated and compared to normative data, used as a cut–off for obvious reported difficulties.
Study participants were contacted by mail including the FTF in Swedish. After two months, a reminder was sent out to the non–responding families, and later a final request.
A paediatric neurologist with no information on delivery mode reviewed the unidentified results by group (</≥90th percentile) for each domain to account for non-random misclassification. The results were transferred to 5–15.org for calculation.

Data acquisition
Maternal, obstetric, and neonatal characteristics were collected from computerized delivery and neonatal charts (Obstetrix®).
Registered ICD–10 diagnoses for the participating children were acquired from the national patient register at the Swedish National Board of Health and Welfare (SNBHW) until 2021. Included ICD–10 codes were F70–73, F78–79 (mental retardation), F80–84, F88.9 and F89.9 (disorders of the psychological development), F90–95, F98–99 (behavioural and emotional disorders), R48 (dyslexia and other symbolic dysfunctions), G80 (cerebral palsy), and G40 and G41 (episodic and paroxysmal disorders). The Swedish national patient register includes information on inpatient care since 1964 and outpatient specialist care since 2001. The validity of most diagnoses, including psychiatric disorders, has proven to be high.[32]
Maternal educational level was collected from the longitudinal integration data base for health insurance and market studies (LISA), Statistics Sweden (Statistiska centralbyrån, SCB). The SUN2000 classification (Svensk UtbildningsNomenklatur) is divided into categories: 0) preschool education; 1) pre– high school education <9 years; 2) pre– high school education 9–10 years; 3) high school education; 4) post– high school education <2 years; 5) post– high school education ≥2 years, and 6) doctoral studies/PhD. A subdivision of post– high school studies yes/no was done (levels 4–6).

Outcomes
The primary outcome was the FTF score for each main domain according to delivery mode. Secondary outcomes were the incidence of scoring ≥90th percentile in ≥2 main domains in the FTF for each delivery mode, and the incidence of an ICD–10 paediatric neurodevelopmental diagnosis.

Statistical analysis

https://mc.manuscriptcentral.com/bmjpo

Confidential: For Review Only

IBM® SPSS® Statistics (version 27) was used reporting 95% confidence intervals and p-values. Descriptive statistics are presented as mean ± standard deviation (SD), median (min–max), and numbers and percentages. The Chi square test was used for dichotomous data. Independent samples Student t-test or Wilcoxon rank sum tests were used for continuous variables. Univariate regression analyses were performed comparing: 1) VAD vs. SVD; 2) SSCD vs. SVD and 3) VAD vs. SSCD, with the last one serving as the reference group for each main domain in the FTF scores.
To assist in choosing the variables in the multivariate logistic regression analyses, a Directed Acyclic Graph (DAG) was designed, investigating potential confounders in the relationship between the exposure (delivery mode) and outcome (response in the FTF) (figure S1). The variables finally included in the analyses were parity, maternal educational level, gestational length (in days), the indication OFHR for the comparison VAD vs SSCD, and the use of oxytocin in the comparison VAD vs SVD and SSCD vs SVD.

Public and Patient Involvement statement
There was no public or patient involvement. Written consent was signed on the front page of the FTF questionnaire.

Results

The annual delivery rate during the study period was 4500 at Karolinska University Hospital Huddinge and 4000 at Solna, including 10 % emergency caesarean sections and 7-9% VADs (50% at outlet station and 7.1% failed extractions). Both delivery wards are managed by the same organization.

1521 families received the FTF questionnaire. After exclusion (Figure 1), 1455 families were invited to participate (VAD n =440, SVD n =844, and SSCD n =171). The response rate was 58% in the VAD group (n =253), 29% in the SVD group (n =247) and 50% in the SSCD group (n =86).

The descriptive analysis on responders and non-responders in the VAD group (table S1) showed no differences except more mid-station VADs in the non-response group (71% vs 59%).

A descriptive analysis according to delivery mode is shown in Table 1.

Table 1: Descriptive analysis according to delivery mode

	VAD (n =253)	SVD (n =247)	SSCD (n =86)	p-value
**Maternal characteristics**				
Nulliparous[1,4]	208 (82%)	151 (61%)	67 (78%)	<0.001[a*]<br><0.01[b*]<br>0.38[c]
Previous caesarean[1,4]	23 (9%)	4 (2%)	11 (13%)	<0.001[a*]<br><0.001[b*]<br>0.32[c]
Post- high school education[1,4]	166 (66%)	185 (75%)	61 (71%)	<0.05[a*]<br>0.5[b]<br>0.4[c]
BMI (kg/m²)[3,6]	23 (18-50)	23 (18-35)	23 (18-37)	0.42[a]<br><0.01[b*]<br><0.05[c*]

Confidential: For Review Only

Gestational length, days[3,6]	281 (261-295)	279 (259-294)	281 (259-295)	<0.001[a*]<br><0.001[b*]<br><0.05[c*]
First stage of labour, hours[2,5]	8.8 (±4.6)	4.2 (±3.7)	8.4 (±5.2)	<0.001[a*]<br><0.001[b*]<br>0.53[c]
Second stage of labour, hours[3,6]	2 (0-6.5)	0.5 (0-5)	5 (0-17)	<0.001[a*]<br><0.001[b*]<br><0.001[c*]
**Delivery characteristics**				
Indication OFHR	108 (43%)		22 (26%)	<0.01[c*]
Indication dystocia	145 (57%)		60 (74%)	
Epidural[1,4]	183 (72%)	78 (32%)	69 (80%)	<0.001[a*]<br><0.001[b*]<br>0.2[c]
Oxytocin[1,4]	242 (96%)	99 (40%)	83 (97%)	<0.001[a*]<br><0.001[b*]<br>0.7[c]
Position OAP[1,4]	221 (90%)	237 (96%)	61 (71%)	<0.05[a*]<br><0.001[b*]<br><0.001[c*]
Shoulder dystocia[1,4]	11 (4%)	0	-	0.001[a*]
**Perinatal characteristics**				
Birth weight, g[2,5]	3668 (±447)	3578 (±429)	3836 (±494)	<0.05[a*]<br><0.001[b*]<br><0.01[c*]
Gender male[1,4]	115 (46%)	119 (48%)	36 (42%)	0.5[a]<br>0.3[b]<br>0.6[c]
NICU admission[1,4]	40 (16%)	1 (0.4%)	5 (6%)	<0.001[a*]<br>0.001[b*]<br><0.05[c*]
pH a. umbilicalis<7.00[1,4]	4 (2%)	0	0	<0.05[a*]<br>0.2[c]
Apgar<7 at 5 min[1,4]	9 (4%)	0	0	<0.01[a*]<br>0.08[c]

*Statistically significant at p-value <0.05. *a: VAD vs. SVD, b: SSCD vs. SVD, c: VAD vs. SSCD.* [1]n (%), [2]mean± sd, [3]median (min-max), [4]Chi[2] test, [5]Student T-test, [6]Wilcoxon rank sum test. BMI: body mass index, OFHR: ominous foetal heart rate, OAP: occipital-anterior position, NICU: neonatal intensive care unit. VAD: vacuum-assisted delivery, SVD: spontaneous vaginal delivery, SSCD: second stage caesarean delivery.

Out of all VADs, 13% failed (n =33) and were delivered by subsequent delivery modes (forceps n=8, SSCD n=17 and forceps plus SSCD n=8). The failed VADs were included in the VAD group.

The Five-to-Fifteen questionnaire

A basic characteristics comparison between children with obvious difficulties in one or more domains according to the answers in the FTF (≥90th percentile) and children without difficulties (<90th percentile), according to normative data, was performed for each delivery mode. For all delivery modes, there were statistically significantly more women with a post-high school education in the group with children scoring <90th percentile. Apart from this, there were more subjectively heavy extractions among children scoring ≥90th percentile born after VAD (p-value <0.05). In the SSCD group there were more males among children scoring <90th percentile (p-value <0.01). Table 2 presents the age distribution of the children delivered by

Confidential: For Review Only

each delivery mode. The FTF was assessed in 2017 in the VAD group, in 2020 in the SVD group, and in 2019-2020 in the SSCD group.

Table 2: Age distribution among the children for each delivery mode

Age (years)	VAD (n =253)	SVD (n =247)	SSCD (n =86)
6y	-	-	11 (13%)
7y	50 (20%)	62 (25%)	13 (15%)
8y	82 (32%)	88 (36%)	16 (19%)
9y	104 (41%)	81(33%)	8 (9%)
10y	17 (7%)	16 (6%)	12 (14%)
11y	-	-	8 (9%)
12y	-	-	17 (20%)
13y	-	-	1 (1%)
Mean age at FTF completion	8 (±0.9)	8 (±0.9)	9 (±2.2)

N (%). VAD: vacuum-assisted delivery, SVD: spontaneous vaginal delivery, SSCD: second stage caesarean delivery, FTF: Five-to-Fifteen Questionnaire

The univariate regression analyses are presented in Table 3. There was a higher proportion of children scoring to have problems linked to motor skills and perception in the VAD group compared to the SVD group. The same results were seen when comparing SSCD to SVD. There were no calculated differences between the VAD and SSCD groups. In the multivariate logistic regression analysis the calculated aOR remained increased for problems with motor skills in children delivered with a VAD or SSCD compared to children delivered with a SVD.

Table 3: Number and percentages of children scoring ≥90th percentile in the FTF per main domain; a univariate and multivariate logistic regression analysis

Main domain	VAD n/total (%)	SVD n/total (%)	SSCD n/total (%)	OR (95% CI)	aOR (95% CI)
Motor skills	47/253 (19%)	23/246 (9%)	22/86 (26%)	2.2 (1.3- 3.8)[a]	2.2 (1.1- 4,7)[a]
				3.3 (1.8-6.4)[b]	3.2 (1.3-7.5)[b]
				0.7 (0.4-1.2)[c]	0.6 (0.3-1.1)[c]
Executive functions	40/253 (16%)	34/247 (14%)	10/86 (12%)	1.2 (0.7-1.9)[a]	1.5 (0.8-3.1)[a]
				0.8 (0.4-1.8)[b]	1.2 (0.5-3.2)[b]
				0.8 (0.4-1.8)[c]	1.2 (0.5-2.5)[c]
Perception	42/253 (17%)	26/247 (11%)	18/86 (21%)	1.7 (1.002-2.9)[a]	1.9 (0.9-4.1)[a]
				2.3 (1.2-4.4)[b]	1.9 (0.8-4.7)[b]
				0.8 (0.4-1.4)[c]	0.6 (0.3-1.2)[c]
Memory	29/253 (11%)	26/247 (11%)	5/86 (6%)	1.1 (0.6-1.9)[a]	1.1 (0.5-2.4)[a]
				0.5 (0.2-1.4)[b]	0.5 (0.1-1.6)[b]
				2.1 (0.8-5.6)[c]	1.9 (0.7-5.1)[c]
Language	31/253 (12%)	22/247 (9%)	6/86 (7%)	1.4 (0.8-2.5)[a]	1.7 (0.7-3.8)[a]
				0.8 (0.3-2)[b]	0.6 (0.2-1.8)[b]

Confidential: For Review Only

				1.9 (0.8-4.6)[c]	1.7 (0.7-4.2)[c]
Learning	30/195 (15%)	23/192 (12%)	7/62 (11%)	1.3 (0.8-2.4)[a]<br>0.9 (0.4-2.3)[b]<br>1.4 (0.6-3.4)[c]	1.6 (0.7-3.5)[a]<br>0.7 (0.2-2.2)[b]<br>1.2 (0.7-3.1)[c]
Social skills	30/252 (12%)	19/246 (8%)	6/81 (7%)	1.6 (0.9-3)[a]<br>0.9 (0.4-2.5)[b]<br>1.7 (0.7-4.2)[c]	1.2 (0.5-2.6)[a]<br>0.6 (0.2-1.9)[b]<br>1.6 (0.6-4.1)[c]
Emotional/ Behavioural problems	45/253 (18%)	29/247 (12%)	13/86 (15%)	1.6 (0.9-2.7)[a]<br>1.3 (0.7-2.7)[b]<br>1.2 (0.6-2.4)[c]	1.4 (0.7-2.8)[a]<br>1.4 (0.5-3.4)[b]<br>1.1 (0.6-2.2)[c]

a: VAD vs. SVD, b: SSCD vs. SVD, c: VAD vs. SSCD. FTF: Five-to-Fifteen questionnaire. VAD: vacuum-assisted delivery, SVD: spontaneous vaginal delivery, SSCD: second stage caesarean delivery. OR: odds ratio. The multivariate logistic regression analysis shows adjusted OR for parity, maternal educational level, gestational length, oxytocin usage in VAD vs. SVD and SSCD vs. SVD, and indication OFHR in VAD vs. SSCD. FTF: Five-to-Fifteen questionnaire. VAD: vacuum-assisted delivery, SVD: spontaneous vaginal delivery, SSCD: second stage caesarean delivery

There were significantly more children with obvious difficulties in two or more domains in the VAD and SSCD groups compared to the SVD group (Table 4).

Table 4: Number and percentages of children scoring ≥90th percentile in the FTF in ≥2 domains; a univariate logistic regression analysis

	VAD n/total (%)	SVD n/total (%)	SSCD n/total (%)	OR (95%CI)
≥2 domains	65/87 (75%)	37/73 (49%)	22/31 (71%)	1.96 (1.3-3.1)[a]<br>1.95 (1.1-3.6)[b]<br>1.00 (0.6-1.8)[c]

*Statistically significant at p-value<0.05. *a: VAD vs. SVD b: SSCD vs. SVD c: VAD vs. SSCD.* FTF: Five-to-Fifteen questionnaire, VAD: vacuum-assisted, SVD: spontaneous vaginal delivery, SSCD: second stage caesarean delivery

The incidence of the diagnoses acquired from the SNBHW are presented in Table 5. There are more children with an attention deficit/hyperactivity disorder in the VAD group compared to the other two delivery modes. Furthermore, children born by VAD had a higher incidence of neurodevelopmental diagnoses than children born by SVD.

Table 5 Children´s diagnoses according to ICD-10

	VAD (n =253)	SVD (n =247)	SSCD (n =86)	Unadjusted OR	95% CI
Communication disorders (F80)	4 (2%)	1 (0.4%)	0	3.9[a]<br>1[b]<br>3.1[c]	0.4 – 36[a]<br>0.04 – 26[b]<br>0.2 – 59[c]
Specific developmental disorder of motor function (F829)	2 (0.8%)	0	0	4.9[a]<br>–<br>1.7[c]	0.2 – 103[a]<br>–<br>0.1 – 36[c]
Autism spectrum disorders (F84)	12 (5%)	6 (2%)	0	2[a]<br>0.2[b]<br>8.9[c]	0.7 – 5[a]<br>0.01 – 4[b]<br>0.5 – 153[c]

Confidential: For Review Only

Unspecified disorder of psychological development (F899)	0	0	1 (1%)	–<br>8.7[b]<br>0.1[c]	–<br>0.4 – 215[b]<br>0.01 – 3[c]
Attention deficit hyperactivity disorder (F90)	23 (9%)	8 (3%)	1 (1%)	3[a]<br>0.4[b]<br>8.5[c]	1.3 – 7[a]<br>0.04 – 3[b]<br>1.1 – 64[c]
Oppositional defiant disorder (F913)	1 (0.4%)	0	0	2.9[a]<br>–<br>1[c]	0.1 – 73[a]<br>–<br>0.04 – 26[c]
Tic disorders (F95)	2 (1%)	0	0	4.9[a]<br>–<br>1.7[c]	0.2 – 103[a]<br>–<br>0.1 – 36[c]
Enuresis (F980)	4 (2%)	4 (2%)	1 (1%)	0.9[a]<br>0.7[b]<br>1.4[c]	0.2 – 4[a]<br>0.1 – 7[b]<br>0.1 – 12[c]
Encopresis (F981)	1 (0.4%)	0	3 (3%)	2.9[a]<br>21[b]<br>0.1[c]	0.1 – 73[a]<br>1.1 – 406[b]<br>0.01 – 1.1[c]
Developmental and emotional disorders (F98)	2 (0.8%)	0	1 (1%)	4.9[a]<br>8.7[b]<br>0.7[c]	0.2 – 103[a]<br>0.4 – 215[b]<br>0.1 – 8[c]
Unspecified mental disorder (F999)	3 (1%)	2 (0.8%)	0	1.5[a]<br>0.6[b]<br>2.4[c]	0.2 – 9[a]<br>0.03 – 12[b]<br>0.1 – 47[c]
Epilepsy (G40)	1 (0.4%)	2 (0.8%)	1 (1%)	0.5[a]<br>0.4[b]<br>0.3[c]	0.04 – 5[a]<br>0.1 – 16[b]<br>0.02 – 6[c]
Neurodevelopmental diagnosis	36 (14%)	15 (6%)	7 (8%)	2.6[a]<br>1.4[b]<br>1.9[c]	1.4 – 5[a]<br>0.5 – 4[b]<br>0.8 – 4[c]
Child with ≥2 neurodevelopmental diagnoses	20 (8%)	6 (2%)	2 (2%)	3.5[a]<br>0.9[b]<br>3.6[c]	1.4 – 9[a]<br>0.2 – 5[b]<br>0.8 – 16[c]

Unadjusted odds ratio (OR) and 95% confidence interval (95% CI). (Haldane-Anscombe correction used for values of 0). N (%). a*: VAD vs. SVD, b: SSCD vs. SVD, c: VAD vs. SSCD.* VAD: vacuum-assisted delivery, SVD: spontaneous vaginal delivery, SSCD: second stage caesarean delivery.

Discussion

Main findings

According to the results from the studied cohort, low- and mid-station VADs imply increased odds of long-term neurodevelopmental difficulties in motor skills and perception between seven and ten years of age compared to spontaneously delivered children. Similar findings were seen in the group delivered with an SSCD. After adjusting for the proposed confounding variables, the increased odds for difficulties in motor skills after a VAD or an SSCD was maintained. The incidence of attention deficit hyperactivity disorder (ADHD) was higher among children born after a VAD.

Considering the relatively frequent use of VAD in modern obstetrics, it is striking how little is known about the long-term outcomes. According to the current study, this group may be at risk for neurodevelopmental difficulties. The information available does not allow one to determine whether the VAD procedure causes these difficulties, or whether the entire delivery process contributes, since children born after an SSCD were calculated to be at a similar risk. It may be that the complete situation, including the lack of progress in the second stage of labour, and the maternal and foetal conditions, are responsible for the problematic outcome. It appears,

Confidential: For Review Only

however, that children born after VAD might have an increased risk of ADHD, as indicated by the register data. This increased incidence was not observed in the SSCD group.

Strengths and limitations

This is a study with a well-defined cohort of children, investigating long-term outcomes using a validated screening method. By including the registered neurodevelopmental ICD-10 diagnoses at the SNBHW, the value of the study is enhanced.

Even though the validated questionnaire contains a normative reference group, we included a control group with children born via SVD to increase validity. In addition, SSCD group with a foetal head station at level 0 or more, without a prior VAD attempt, is a clinically relevant comparison group. This group had a significantly longer duration of the second stage (a median of five hours) and showed similar results in the FTF as the VAD group. On the other hand, there were more children with neurodevelopmental diagnoses and NICU admissions in the VAD group.

It might be questioned whether parents' estimates of a child's development are objective. However, the FTF is not a diagnostic tool, and a multi-professional assessment is needed to diagnose neurodevelopmental disorders in children and youth. Since the questionnaire was distributed only in the Swedish language, an inevitable selection of parents might exist, but this applies to the whole cohort.
The maternal educational level was included as a proxy for socioeconomic status, since most questionnaires were filled out by mothers. Mothers with higher educational levels more often had children scoring <90th percentile, and mothers in the VAD group had significantly lower educational level than the other two groups. This variable was adjusted for in the multivariate regression analysis.

The low response rate in the SVD group is a limitation and could be attributed to the fact that a control group is less likely to respond. This could potentially lead to selection bias, resulting in parents of children with difficulties being more likely to respond. Alternatively, it may be that parents who experienced a traumatic delivery were less prone to respond. The response analysis in the VAD group did not reveal any differences, except a higher rate of mid-station VADs in the non-respondent group, which is believed to not affect the result, except a possible risk for underestimation of FTF scores.
Including the failed VADs in this group may bias the result by including children with worse outcomes but shows the relevance in the clinical reality. On the other hand, the SSCD group was clinically more uniform for comparison. Since the SSCD group is smaller, differences may be hidden. To remedy the small sample size in the SSCD group, the age range was extended while still suitable for the FTF. We believe this was necessary to try to answer the research question without introducing risks.

Interpretation

The study is observationally designed to screen for neurodevelopmental difficulties rather than to reveal causal relationships. With the use of FTF several neurodevelopmental aspects were possible to study. The results are in contrast with the few existing studies reporting on specific long–term neurodevelopmental aspects,[19-21 23 33] but these have not stratified according to the VAD type, they have smaller cohorts, and did not use validated follow–up methods.

Even though children had motor skill problems according to the FTF, they were not registered with individual motor skill diagnoses. This may rather be revealed in a more complex form i.e., as a comorbidity in ADHD. Whether or not low– and mid-station station VAD or SSCD affect

Confidential: For Review Only

specific parts of the foetal brain resulting in impaired neurodevelopment for different skills, is unknown. Although the etiology of diagnoses such as ADHD is heterogeneous and unclear, it is known to be associated with traumatic brain injury.[34] Apart from this, there is an established association between ADHD and heredity.[35 36] Registered neurodevelopmental diagnoses among parents or siblings were not collected.

Conclusion

Obstructed labour at the second stage in term deliveries is a delicate mission for clinicians. Although interpreted with caution, the significant difference in long-term neurodevelopmental difficulties in children delivered with VAD or SSCD compared to a SVD is worrying and indicates the need for increased knowledge in the field. If additional studies show similar results, it seems necessary to revise the existing guidelines for VAD and SSCD.

Ethical approval Ethical approval was obtained by the Regional Ethical Review Board Stockholm, Sweden (DNR 2014/1860–31, DNR 2017/1411–32, DNR 2019/04880 and DNR 2021/02457).
Funding statement This work was supported by the Stockholm City Council (Agreement for Research and Education of Doctors) (grant number FoUI-953029 and FoUI-955070), CIMED (Centre for Innovative Medicine) (grant number FoUI-954462), Karolinska Institutet and General Maternity Hospital Foundation (Stiftelsen Allmänna BB:s Minnesfond) (grant number N/A).
Competing interest statement All authors have completed the ICMJE uniform disclosure form at http://www.icmje.org/disclosure-of-interest/ and declare: no support from any organisation for the submitted work; no financial relationships with any organisations that might have an interest in the submitted work in the previous three years; no other relationships or activities that could appear to have influenced the submitted work.
Authors' contributions GA and MW designed the study, acquired, analysed, interpreted the data, and wrote and revised the manuscript. SR acquired, analysed, interpreted the data, and wrote and revised the manuscript. KL analysed, interpreted the data, and revised the manuscript. The corresponding author attests that all listed authors meet authorship criteria and that no others meeting the criteria have been omitted.
Acknowledgements The paper is part of Dr. Romero's doctoral thesis.
STROBE checklist see supplementary files
Data sharing statement "The Corresponding Author has the right to grant on behalf of all authors and does grant on behalf of all authors, if the manuscript is accepted, a worldwide licence to the Publishers and its licensees in perpetuity, in all forms, formats and media (whether known now or created in the future), to i) publish, reproduce, distribute, display and store the Contribution, ii) translate the Contribution into other languages, create adaptations, reprints, include within collections and create summaries, extracts and/or, abstracts of the Contribution, iii) create any other derivative work(s) based on the Contribution, iv) to exploit all subsidiary rights in the Contribution, v) the inclusion of electronic links from the Contribution to third party material wherever it may be located; and, vi) licence any third party to do any or all of the above."
Transparency declaration: The lead author (GA) affirms that the manuscript is an honest, accurate, and transparent account of the study being reported; that no important aspects of the study have been omitted and that any discrepancies from the study as planned have been explained.

Confidential: For Review Only

Summary box

What is already known on this topic

- There are very few studies investigating the long–term neurodevelopmental difficulties in children born after a vacuum-assisted delivery, and there is an evident lack of information.
- No differences were reported in previous studies on long-term neurodevelopmental outcomes after vacuum-assisted delivery.
- Several methodological issues have been identified in previous studies, including limited information regarding the vacuum-assisted procedure and the use of non-validated methods for neurodevelopmental screening.

What this study adds

- Children born after low– or mid-station station vacuum-assisted deliveries may be at an increased risk for long–term neurodevelopmental difficulties in comparison to those born after a spontaneous vaginal delivery.
- Similar results were seen in the group of children born after an emergency caesarean delivery at the second stage of labour compared to those born after a spontaneous vaginal delivery.
- Children delivered with a vacuum-assisted delivery may have more attention deficit/hyperactivity disorders than children born after the other two delivery modes.

How this study might affect research, practice or policy

- Considering the frequent use of the VAD and the SSCD in the second stage of labour, the unexpected results of an increased odds of long-term problems in motor skills and perception in these children is striking and worrying.
- These results need to be promptly investigated, preferably in prospective studies. If similar results are seen, the clinical guidelines for these two methods need to be revised.

Confidential: For Review Only

References

1. Malmstrom T. Vacuum extractor, an obstetrical instrument. *Acta Obstet Gynecol Scand Suppl* 1954;33(4):1-31. [published Online First: 1954/01/01]
2. Martin Ja Fau - Hamilton BE, Hamilton Be Fau - Osterman MJK, Osterman Mjk Fau - Driscoll AK, et al. Births: Final Data for 2017. (1551-8922 (Print))
3. The Swedish National Board of Health and Welfare T. Statistikbas för graviditeter, förlossningar och nyfödda. 2020 [Available from: https://www.socialstyrelsen.se/statistik-och-data/statistik/alla-statistikamnen/graviditeter-forlossningar-och-nyfodda/.
4. NHS. NHS Maternity Statistics, England. England: NHS Digital, 2021.
5. Canadian Institute for Health Information. Assisted delivery rate (overall) among vaginal deliveries, 2022.
6. (RANZCOG) TRNZCoOaG. Instrumental vaginal birth. RANZCOG web page, 2020.
7. Macfarlane AJ, Blondel B, Mohangoo AD, et al. Wide differences in mode of delivery within Europe: risk-stratified analyses of aggregated routine data from the Euro-Peristat study. *Bjog* 2016;123(4):559-68. doi: 10.1111/1471-0528.13284 [published Online First: 2015/03/11]
8. Ekeus C, Hogberg U, Norman M. Vacuum assisted birth and risk for cerebral complications in term newborn infants: a population-based cohort study. *BMC Pregnancy Childbirth* 2014;14:36. doi: 10.1186/1471-2393-14-36 [published Online First: 2014/01/22]
9. Muraca GM, Boutin A, Razaz N, et al. Maternal and neonatal trauma following operative vaginal delivery. *CMAJ* 2022;194(1):E1-E12. doi: 10.1503/cmaj.210841 [published Online First: 2022/01/12]
10. Gebremariam A. Subgaleal haemorrhage: risk factors and neurological and developmental outcome in survivors. *Ann Trop Paediatr* 1999;19(1):45-50. doi: 10.1080/02724939992626 [published Online First: 1999/12/22]
11. Towner DR, Ciotti MC. Operative vaginal delivery: a cause of birth injury or is it? *Clin Obstet Gynecol* 2007;50(3):563-81. doi: 10.1097/GRF.0b013e31811eaa39 [published Online First: 2007/09/01]
12. Singh A, Rathore P. A comparative study of feto-maternal outcome in instrumental vaginal delivery. *J Obstet Gynaecol India* 2011;61(6):663-6. doi: 10.1007/s13224-011-0119-3 [published Online First: 2012/12/04]
13. Hehir MP, Reidy FR, Wilkinson MN, et al. Increasing rates of operative vaginal delivery across two decades: accompanying outcomes and instrument preferences. *Eur J Obstet Gynecol Reprod Biol* 2013;171(1):40-3. doi: 10.1016/j.ejogrb.2013.08.015 [published Online First: 2013/09/04]
14. Muraca GM, Skoll A, Lisonkova S, et al. Perinatal and maternal morbidity and mortality among term singletons following midcavity operative vaginal delivery versus caesarean delivery. *BJOG* 2018;125(6):693-702. doi: 10.1111/1471-0528.14820 [published Online First: 2017/07/12]
15. Walsh CA, Robson M, McAuliffe FM. Mode of delivery at term and adverse neonatal outcomes. *Obstet Gynecol* 2013;121(1):122-8. doi: http://10.1097/AOG.0b013e3182749ac9
10.1097/aog.0b013e3182749ac9 [published Online First: 2012/12/25]

https://mc.manuscriptcentral.com/bmjpo

Confidential: For Review Only

16. Halscott TL, Reddy UM, Landy HJ, et al. Maternal and Neonatal Outcomes by Attempted Mode of Operative Delivery From a Low Station in the Second Stage of Labor. *Obstet Gynecol* 2015;126(6):1265-72. doi: 10.1097/AOG.0000000000001156 [published Online First: 2015/11/10]
17. Vacca A. Reducing the Risks of a Vacuum Delivery. *Fetal Medicine Review* 2006;17 (4):301-15. doi: doi:10.1017/S0965539506001823
18. Murphy DJ, Strachan BK, Bahl R, et al. Assisted Vaginal Birth: Green-top Guideline No. 26. *BJOG* 2020;127(9):e70-e112. doi: 10.1111/1471-0528.16092 [published Online First: 2020/04/30]
19. Ahlberg M, Ekeus C, Hjern A. Birth by vacuum extraction delivery and school performance at 16 years of age. *Am J Obstet Gynecol* 2014;210(4):361 e1-61 e8. doi: 10.1016/j.ajog.2013.11.015 [published Online First: 2013/11/13]
20. Ngan HY, Miu P, Ko L, et al. Long-term neurological sequelae following vacuum extractor delivery. *Aust N Z J Obstet Gynaecol* 1990;30(2):111-4. [published Online First: 1990/05/01]
21. Johanson RB, Heycock E, Carter J, et al. Maternal and child health after assisted vaginal delivery: five-year follow up of a randomised controlled study comparing forceps and ventouse. *Br J Obstet Gynaecol* 1999;106(6):544-9. [published Online First: 1999/07/30]
22. Bjerre I, Dahlin K. The long-term development of children delivered by vacuum extraction. *Dev Med Child Neurol* 1974;16(3):378-81. doi: 10.1111/j.1469-8749.1974.tb03354.x [published Online First: 1974/06/01]
23. Seidman DS, Laor A, Gale R, et al. Long-term effects of vacuum and forceps deliveries. *Lancet* 1991;337(8757):1583-5. [published Online First: 1991/06/29]
24. Mastrolia SA, Wainstock T, Sheiner E, et al. Failed Vacuum and the Long-Term Neurological Impact on the Offspring. *Am J Perinatol* 2017;34(13):1306-11. doi: 10.1055/s-0037-1603507 [published Online First: 2017/06/01]
25. Ayala NK, Schlichting LE, Kole MB, et al. Operative vaginal delivery and third grade educational outcomes. *Am J Obstet Gynecol MFM* 2020;2(4):100221. doi: 10.1016/j.ajogmf.2020.100221 [published Online First: 2020/12/22]
26. ACOG. Operative Vaginal Birth: ACOG Practice Bulletin, Number 219. *Obstet Gynecol* 2020;135(4):e149-e59. doi: 10.1097/AOG.0000000000003764 [published Online First: 2020/03/29]
27. Vladic-Stjernholm YWAA-WI. Instrumental delivery by vacuum extraction: advice for clinical recommendations (in Swedish): Löf 2017 [Available from: https://lof.se/filer/Instrumentell-forlossning-med-sugklocka.pdf.
28. Kadesjo B, Janols LO, Korkman M, et al. The FTF (Five to Fifteen): the development of a parent questionnaire for the assessment of ADHD and comorbid conditions. *Eur Child Adolesc Psychiatry* 2004;13 Suppl 3:3-13. doi: 10.1007/s00787-004-3002-2 [published Online First: 2005/02/05]
29. Trillingsgaard A, Damm D, Sommer S, et al. Developmental profiles on the basis of the FTF (Five to Fifteen) questionnaire-clinical validity and utility of the FTF in a child psychiatric sample. *Eur Child Adolesc Psychiatry* 2004;13 Suppl 3:39-63. doi: 10.1007/s00787-004-3006-y [published Online First: 2005/02/05]
30. Korkman M, Jaakkola M, Ahlroth A, et al. Screening of developmental disorders in five-year-olds using the FTF (Five to Fifteen) questionnaire: a validation study. *Eur Child*

https://mc.manuscriptcentral.com/bmjpo

Confidential: For Review Only

Adolesc Psychiatry 2004;13 Suppl 3:31-8. doi: 10.1007/s00787-004-3005-z [published Online First: 2005/02/05]

31. Bohlin G, Janols LO. Behavioural problems and psychiatric symptoms in 5-13 year-old Swedish children-a comparison of parent ratings on the FTF (Five to Fifteen) with the ratings on CBCL (Child Behavior Checklist). *Eur Child Adolesc Psychiatry* 2004;13 Suppl 3:14-22. doi: 10.1007/s00787-004-3003-1 [published Online First: 2005/02/05]
32. Ludvigsson JF, Andersson E, Ekbom A, et al. External review and validation of the Swedish national inpatient register. *BMC Public Health* 2011;11:450. doi: 10.1186/1471-2458-11-450 [published Online First: 2011/06/11]
33. Nilsen ST. Boys born by forceps and vacuum extraction examined at 18 years of age. *Acta Obstet Gynecol Scand* 1984;63(6):549-54. doi: 10.3109/00016348409156718 [published Online First: 1984/01/01]
34. Asarnow RF, Newman N, Weiss RE, et al. Association of Attention-Deficit/Hyperactivity Disorder Diagnoses With Pediatric Traumatic Brain Injury: A Meta-analysis. *JAMA Pediatr* 2021;175(10):1009-16. doi: 10.1001/jamapediatrics.2021.2033 [published Online First: 2021/07/13]
35. Larsson H, Chang Z, D'Onofrio BM, et al. The heritability of clinically diagnosed attention deficit hyperactivity disorder across the lifespan. *Psychol Med* 2014;44(10):2223-9. doi: 10.1017/S0033291713002493 [published Online First: 2013/10/11]
36. Faraone SV, Larsson H. Genetics of attention deficit hyperactivity disorder. *Mol Psychiatry* 2019;24(4):562-75. doi: 10.1038/s41380-018-0070-0 [published Online First: 2018/06/13]

Confidential: For Review Only

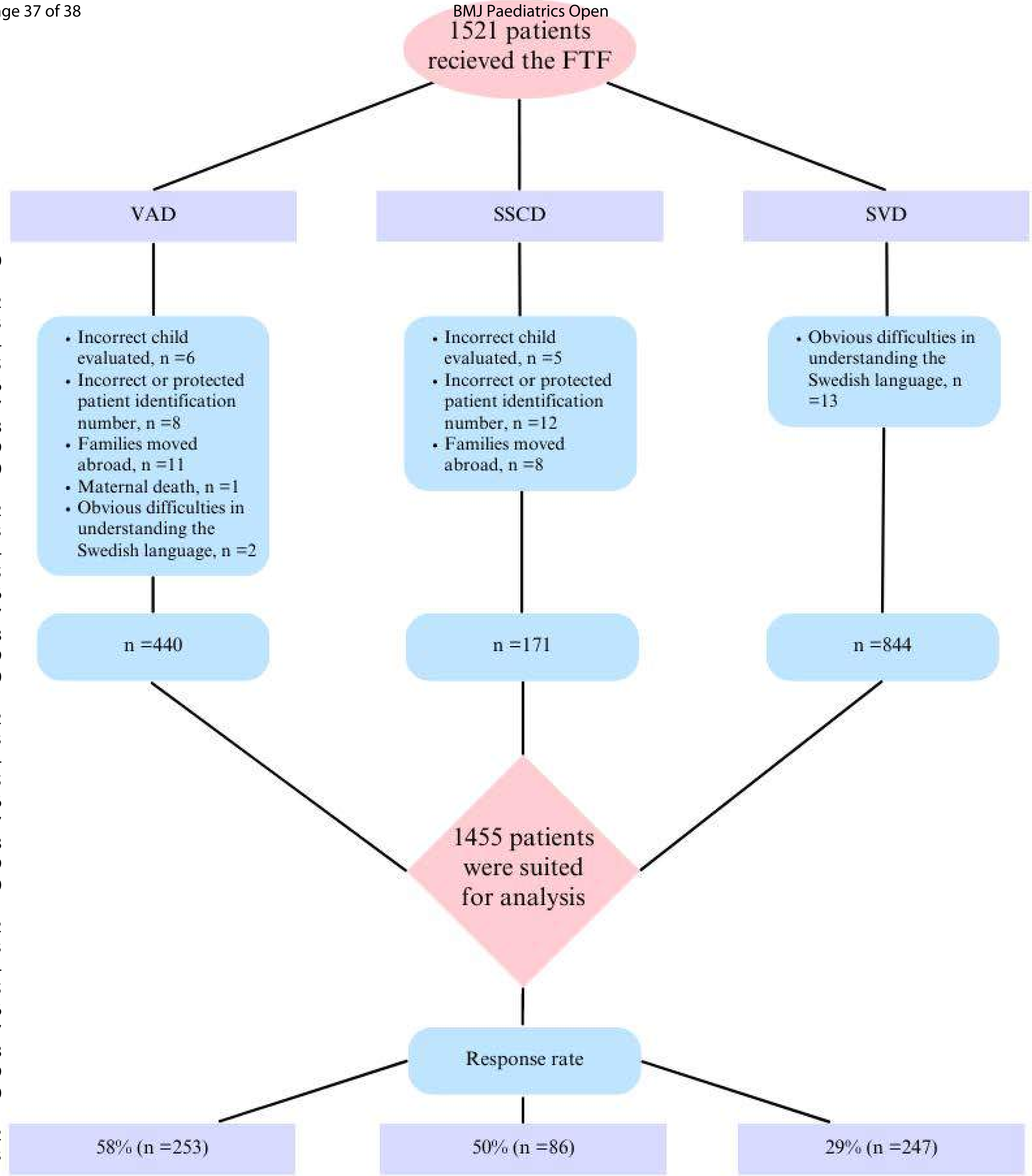

Figure 1: Study flowchart

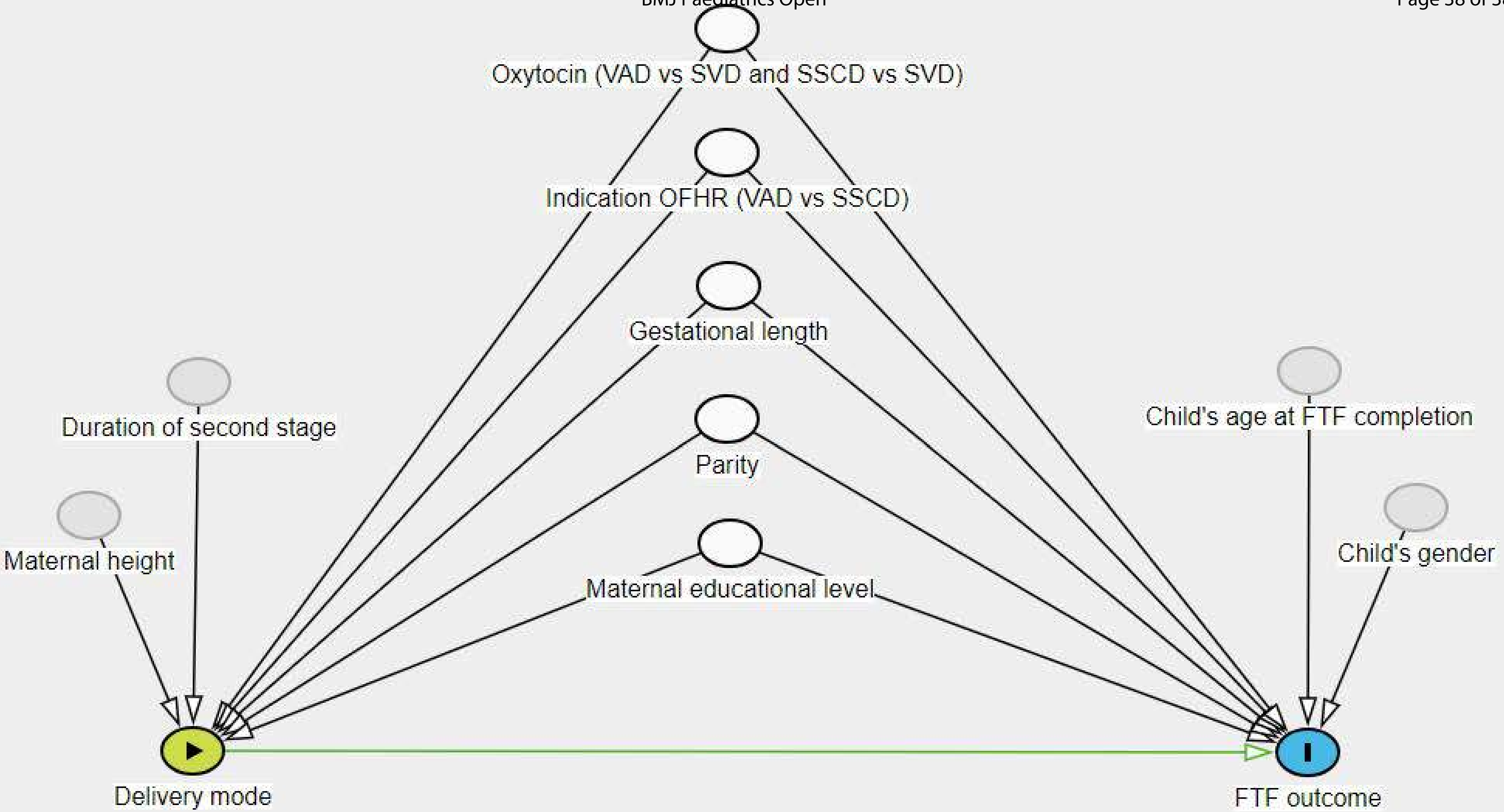

Figure S1: Directed Acyclic Graph (DAG)

Table S1: Descriptive analysis of respondents and non-respondents in the vacuum-assisted delivery group

	FTF (n =253)	No FTF (n =187)	p-value
**Maternal characteristics**			
BMI (kg/m^2)[3]	23 (16-50)[§]	23 (17-39)[†]	0.82[6]
Nulliparous[1]	208 (82%)	146 (78%)	0.28[4]
Previous caesarean[1]	23 (9%)	20 (11%)	0.58[4]
Gestational length, days[2]	281±8	282±9	0.30[5]
**Delivery characteristics**			
First stage of labour, hours[2]	9±5	9±5	0.56[5]
Second stage of labour, hours[2]	2±2	2±2	0.22[5]
Epidural[1]	175 (72%)	135 (78%)	0.22[4]
Oxytocin[1]	242 (96%)	172 (92%)	0.11[4]
Indication OFHR[1]	108 (43%)	89 (48%)	0.31[4]
Station Mid[1]	148 (59%)	133 (71%)	<0.05[4*]
Number of pulls, n[3]	4 (1-10)	4 (1-13)	0.4[6]
Time during vacuum extraction, min[3]	7 (1-31)	7 (1-29)	0.94[6]
Position OAP[1]	221 (87%)	159 (85%)	0.48[4]
Subjectively heavy extraction[1]	58 (25%)	48 (28%)	0.44[4]
Failed vacuum extraction[1]	34 (13%)	33 (18%)	0.23[4]
Cup detachment[1]	39 (15%)	29 (16%)	0.98[4]
Shoulder dystocia[1]	11 (4%)	13 (7%)	0.23[4]
**Perinatal characteristics**			
Birth weight, g[2]	3668 ± 447	3641 ± 455	0.54[5]
Gender male[1]	138 (55%)	118 (63%)	0.07[4]
pH<7.00[1]	4 (2%)[‡]	0[ⱡ]	0.09[4]
Apgar<7 at 5 min[1]	9 (4%)	9 (5%)	0.51[4]
Severe outcome[1] (ICH, HIE, seizures, subgaleal haematoma)	4 (2%)	5 (3%)	0.42[4]

*Statistically significant at p-value <0.05. [1]n (%), [2]mean±sd, [3]median (min–max), [4]Chi^2 test, [5]Student T-test, [6]Wilcoxon rank sum test. [§]missing data 11%, [†]missing data 10%, [‡]missing data 18%, [ⱡ]missing data 19%. FTF: Five- to Fifteen questionnaire. BMI: body mass index, OFHR: ominous foetal heart rate, OAP: occipital-anterior position, ICH: intracranial haemorrhage, HIE: hypoxic ischemic encephalopathy

Confidential: For Review Only

BMJ Paediatrics Open

Long-term neurodevelopmental outcome in children born after vacuum-assisted delivery compared to second stage caesarean delivery and spontaneous vaginal delivery - a cohort study

Journal:	*BMJ Paediatrics Open*
Manuscript ID	bmjpo-2023-002048.R2
Article Type:	Original research
Date Submitted by the Author:	06-Sep-2023
Complete List of Authors:	Romero, Stefhanie; Karolinska Institute Department of Clinical Science Intervention and Technology, Clinical science, intervention and technology; Karolinska University Hospital, Pregnancy Care and Delivery<br>Lindström, Katarina; Karolinska Institute Department of Clinical Science Intervention and Technology, Clinical Science Intervention and Technology; Karolinska University Hospital, Neuropaediatrics<br>Listermar, Johanna; Karolinska Institute<br>Westgren, Magnus; Karolinska Institute Department of Clinical Science Intervention and Technology, Clinical Science Intervention and Technology<br>Ajne, Gunilla; Karolinska Institute Department of Clinical Science Intervention and Technology, Clinical Science Intervention and Technology; Karolinska University Hospital, Pregnancy Care and Delivery
Keywords:	Psychology, Pathology, Neonatology, Epidemiology, Adolescent Health

SCHOLARONE™
Manuscripts

I, the Submitting Author has the right to grant and does grant on behalf of all authors of the Work (as defined in the below author licence), an exclusive licence and/or a non-exclusive licence for contributions from authors who are: i) UK Crown employees; ii) where BMJ has agreed a CC-BY licence shall apply, and/or iii) in accordance with the terms applicable for US Federal Government officers or employees acting as part of their official duties; on a worldwide, perpetual, irrevocable, royalty-free basis to BMJ Publishing Group Ltd ("BMJ") its licensees and where the relevant Journal is co-owned by BMJ to the co-owners of the Journal, to publish the Work in this journal and any other BMJ products and to exploit all rights, as set out in our licence.

The Submitting Author accepts and understands that any supply made under these terms is made by BMJ to the Submitting Author unless you are acting as an employee on behalf of your employer or a postgraduate student of an affiliated institution which is paying any applicable article publishing charge ("APC") for Open Access articles. Where the Submitting Author wishes to make the Work available on an Open Access basis (and intends to pay the relevant APC), the terms of reuse of such Open Access shall be governed by a Creative Commons licence – details of these licences and which Creative Commons licence will apply to this Work are set out in our licence referred to above.

Other than as permitted in any relevant BMJ Author's Self Archiving Policies, I confirm this Work has not been accepted for publication elsewhere, is not being considered for publication elsewhere and does not duplicate material already published. I confirm all authors consent to publication of this Work and authorise the granting of this licence.

Confidential: For Review Only

Long-term neurodevelopmental outcome in children born after vacuum-assisted delivery compared to second stage caesarean delivery and spontaneous vaginal delivery: a cohort study

Stefhanie Romero[1,2], MD PhD, Katarina Lindström[3,4], MD PhD, Johanna Listermar, Medical student[5], Magnus Westgren[1], MD PhD, Gunilla Ajne[1,2], MD PhD

[1]Division of Obstetrics and Gynaecology, CLINTEC, Karolinska Institutet, Stockholm, 171 77, Sweden
[2] Pregnancy Care & Delivery, Karolinska University Hospital, Stockholm, 141 86, Sweden,
[3]Division of Paediatrics, CLINTEC, Karolinska Institutet, Stockholm, 171 77, Sweden
[4]Neuropediatrics, Karolinska University Hospital, Stockholm, 141 86, Sweden
[5]Karolinska Institutet, Stockholm, 171 77, Sweden
Correspondence to: Stefhanie Romero, Pregnancy Care & Delivery, B76, Karolinska University Hospital, Stockholm, 141 86, Sweden. stefhanie.romero@ki.se.

Confidential: For Review Only

Abstract

Objective To evaluate long-term neurodevelopment in children born after low-or mid-station vacuum-assisted delivery (VAD) compared to children delivered by second stage cesarean delivery (SSCD) or spontaneous vaginal delivery (SVD).

Design Cross sectional cohort study

Setting Two delivery wards, Karolinska University Hospital, Sweden

Patients 253 children born by low– or mid–station VAD, 247 children born after an SVD, and 86 children born via an SSCD accepted to participate.

Interventions The Five-to-Fifteen questionnaire was used as a validated screening method for neurodevelopmental difficulties, assessed by parents.

Main outcomes measures Results in the Five-to-Fifteen questionnaire. In addition, registered neurodevelopmental ICD-10 diagnoses were collected. Regression analyses estimated associations between delivery modes.

Results Children born after VAD exhibited an increased rate of long–term neurodevelopmental difficulties in motor skills (OR 2.2, 95%CI 1.3 to 3.8) and perception (OR 1.7, 95%CI 1.002 to 2.9) compared to SVD. Similar findings were seen in the group delivered with an SSCD compared to SVD (motor skills: OR 3.3, 95%CI 1.8 to 6.4 and perception: OR 2.3, 95%CI 1.2 to 4.4). The increased odds for motor skills difficulties after VAD and SSCD remained after adjusting for proposed confounding variables. There were significantly more children in the VAD group with registered neurodevelopmental ICD–10 diagnoses such as attention deficit/hyperactivity disorders.

Conclusions The differences in long–term neurodevelopmental difficulties in children delivered with a VAD or SSCD compared to SVD in the present study, indicate the need for increased knowledge in the field to optimize the management of second stage of labor.

Key message

What is already known on this topic

- There is an evident lack of information on the long–term neurodevelopmental difficulties in children born after a vacuum-assisted delivery.

What this study adds

- Children born after low– or mid-station station vacuum-assisted deliveries and second stage caesarean deliveries may be at an increased odds of long–term neurodevelopmental difficulties in comparison to those born after a spontaneous vaginal delivery.
- Children delivered with a vacuum-assisted delivery may have more attention deficit/hyperactivity disorders than the other two delivery groups.

How this study might affect research, practice or policy

- These striking results need to be promptly investigated, preferably in prospective studies, with a revision of the clinical guidelines.

Introduction

The vacuum extractor is used in cases of dystocia or foetal distress during the second stage of labour.[1] The incidence of vacuum-assisted delivery (VAD) varies worldwide, representing up to 3% among all vaginal births in the USA,[2] and 5–10% in Europe, Canada, and Australia/New Zealand.[3-7]
Serious foetal complications are rare after VAD, but appears to have a higher rate of intracranial haemorrhage (0.1-0.8%)[8] and subgaleal hematomas (0.4 - 2.5%)[9] compared to spontaneous vaginal delivery (SVD).[10, 11] Results are not consistent when comparing VAD to other instrumental delivery modes, including caesarean delivery at the second stage of labour (SSCD).[9, 12, 13] Some studies indicate an increased risk of birth trauma in the VAD group,[14] while others report no differences in severe neonatal outcomes.[15, 16] The most appropriate comparison to VAD is SSCD since both are optional delivery methods to a SVD at this point.
VAD is classified according to foetal head station in the birth canal into mid, low and outlet stations, the last of which is considered a low risk VAD.[17, 18] Previous research has not always taken this stratification into account, which could be one reasons behind the divergent results.

Considering the large number of children exposed to VAD, there is limited information regarding long–term outcomes. Existing studies have not revealed any significant differences in neurodevelopment outcome.[19-25]

The aim of the present study was to study neurodevelopment in children delivered at term by low- or mid-station station VAD compared to children born after SSCD and SVD.

Confidential: For Review Only

Methods

Study design and study population

This was a cross sectional cohort study, including all women delivered with a low– or mid-station VAD at Karolinska University Hospital Huddinge, Sweden between 2007 and 2009 (n =493). Each individual extraction protocol was examined to confirm the classification of foetal station.[6, 18, 26] Exclusion criteria were outlet station VADs, preterm delivery <37+0 gestational weeks (n=19), BMI <18, and multiple pregnancies (n=6), with a final number of 468 children.

The SVD group consisted of women and their children delivered at Karolinska University Hospital Huddinge. This group was included three years after the VAD group but matched by calendar date and time of birth (2010-2012, n =870). After applying the exclusion criteria, the final group consisted of 869 children. In addition, we identified all cases of SSCD with foetal heads at least at station 0 by scrutinizing the medical records. As this delivery mode was expected to be a rarer event, we included all women at both delivery wards at the Karolinska University Hospital, Huddinge and Solna, between 2007 and 2014 (n=184).

The VAD followed national and international guidelines consisting of limiting the procedure to 20 minutes, a maximum of two cup–detachments and six pulls.[6, 18, 26, 27] The use of a Bird metal cup (50mm) and a vacuum pressure of 80kPa was standard. Both the VAD and the SSCD procedures were exclusively carried out by physicians. SVDs were managed by a midwife, according to routine procedures in the Swedish Health Care system.
A predefined response analysis was included in the study protocol for the VAD group.

Long-term follow-up

The Five–to Fifteen Questionnaire (FTF) was used as a primary long–term follow up. It is a validated screening method for behavioural and neurodevelopmental difficulties.[28] It is aimed at parents, covering different aspects of the child´s functioning. It is subdivided into three age groups (6–8 years, 9–12 years and 13–15 years) and presented for boys and girls of a Swedish norm group.[28-31]
It comprises 181 items divided into eight main domains (motor skills, executive functions, perception, memory, language, learning, social skills, and emotional/behavioural problems). The 90th percentile is calculated and compared to normative data, used as a cut–off for obvious reported difficulties.
Study participants were contacted by mail including the FTF in Swedish. After two months, a reminder was sent out to the non–responding families, and later a final request.
A paediatric neurologist with no information on delivery mode reviewed the unidentified results by group (</≥90th percentile) for each domain to account for non-random misclassification. The results were transferred to 5–15.org for calculation.

Data acquisition

Maternal, obstetric, and neonatal characteristics were collected from computerized delivery and neonatal charts (Obstetrix®).
Registered ICD–10 diagnoses for the participating children were acquired from the national patient register at the Swedish National Board of Health and Welfare (SNBHW) until 2021. Included ICD–10 codes were F70–73, F78–79 (mental retardation), F80–84, F88.9 and F89.9 (disorders of the psychological development), F90–95, F98–99 (behavioural and emotional disorders), R48 (dyslexia and other symbolic dysfunctions), G80 (cerebral palsy), and G40 and G41 (episodic and paroxysmal disorders). The Swedish national patient register includes

https://mc.manuscriptcentral.com/bmjpo

information on inpatient care since 1964 and outpatient specialist care since 2001. The validity of most diagnoses, including psychiatric disorders, has proven to be high.[32]
Maternal educational level was collected from the longitudinal integration data base for health insurance and market studies (LISA), Statistics Sweden (Statistiska centralbyrån, SCB). The SUN2000 classification (Svensk UtbildningsNomenklatur) is divided into categories: 0) preschool education; 1) pre– high school education <9 years; 2) pre– high school education 9–10 years; 3) high school education; 4) post– high school education <2 years; 5) post– high school education ≥2 years, and 6) doctoral studies/PhD. A subdivision of post– high school studies yes/no was done (levels 4–6).

Outcomes
The primary outcome was the FTF score for each main domain according to delivery mode. Secondary outcomes were the incidence of scoring ≥90th percentile in ≥2 main domains in the FTF for each delivery mode, and the incidence of an ICD–10 paediatric neurodevelopmental diagnosis.

Statistical analysis
IBM® SPSS® Statistics (version 27) was used reporting 95% confidence intervals and p-values. Descriptive statistics are presented as mean ± standard deviation (SD), median (min–max), and numbers and percentages. The Chi square test was used for dichotomous data. Independent samples Student t-test or Wilcoxon rank sum tests were used for continuous variables. Univariate regression analyses were performed comparing: 1) VAD vs. SVD; 2) SSCD vs. SVD and 3) VAD vs. SSCD, with the last one serving as the reference group for each main domain in the FTF scores.
To assist in choosing the variables in the multivariable logistic regression analyses, a Directed Acyclic Graph (DAG) was designed, investigating potential confounders in the relationship between the exposure (delivery mode) and outcome (response in the FTF) (figure S1). The variables finally included in the analyses were parity, maternal educational level, gestational length (in days), the indication OFHR for the comparison VAD vs SSCD, and the use of oxytocin in the comparison VAD vs SVD and SSCD vs SVD.

Public and Patient Involvement statement
There was no public or patient involvement. Written consent was signed on the front page of the FTF questionnaire.

Results

The annual delivery rate during the study period was 4500 at Karolinska University Hospital Huddinge and 4000 at Solna, including 10 % emergency caesarean sections and 7-9% VADs (50% at outlet station and 7.1% failed extractions). Both delivery wards are managed by the same organization.

1521 families received the FTF questionnaire. After exclusion (Figure 1), 1455 families were invited to participate (VAD n =440, SVD n =844, and SSCD n =171). The response rate was 58% in the VAD group (n =253), 29% in the SVD group (n =247) and 50% in the SSCD group (n =86).

The descriptive analysis on responders and non-responders in the VAD group (table S1) showed no differences except more mid-station VADs in the non-response group (71% vs 59%).

A descriptive analysis according to delivery mode is shown in Table 1.

Confidential: For Review Only

Table 1: Descriptive analysis according to delivery mode

	VAD (n =253)	SVD (n =247)	SSCD (n =86)	p-value
**Maternal characteristics**				
Nulliparous[1,4]	208 (82%)	151 (61%)	67 (78%)	<0.001[a*]<br><0.01[b*]<br>0.38[c]
Previous caesarean[1,4]	23 (9%)	4 (2%)	11 (13%)	<0.001[a*]<br><0.001[b*]<br>0.32[c]
Post- high school education[1,4]	166 (66%)	185 (75%)	61 (71%)	<0.05[a*]<br>0.5[b]<br>0.4[c]
BMI (kg/m^2)[3,6]	23 (18-50)	23 (18-35)	23 (18-37)	0.42[a]<br><0.01[b*]<br><0.05[c*]
Gestational length, days[3,6]	281 (261-295)	279 (259-294)	281 (259-295)	<0.001[a*]<br><0.001[b*]<br><0.05[c*]
First stage of labour, hours[2,5]	8.8 (±4.6)	4.2 (±3.7)	8.4 (±5.2)	<0.001[a*]<br><0.001[b*]<br>0.53[c]
Second stage of labour, hours[3,6]	2 (0-6.5)	0.5 (0-5)	5 (0-17)	<0.001[a*]<br><0.001[b*]<br><0.001[c*]
**Delivery characteristics**				
Indication OFHR	108 (43%)		22 (26%)	<0.01[c*]
Indication dystocia	145 (57%)		60 (74%)	
Epidural[1,4]	183 (72%)	78 (32%)	69 (80%)	<0.001[a*]<br><0.001[b*]<br>0.2[c]
Oxytocin[1,4]	242 (96%)	99 (40%)	83 (97%)	<0.001[a*]<br><0.001[b*]<br>0.7[c]
Position OAP[1,4]	221 (90%)	237 (96%)	61 (71%)	<0.05[a*]<br><0.001[b*]<br><0.001[c*]
Shoulder dystocia[1,4]	11 (4%)	0	-	0.001[a*]
**Perinatal characteristics**				
Birth weight, g[2,5]	3668 (±447)	3578 (±429)	3836 (±494)	<0.05[a*]<br><0.001[b*]<br><0.01[c*]
Gender male[1,4]	115 (46%)	119 (48%)	36 (42%)	0.5[a]<br>0.3[b]<br>0.6[c]
NICU admission[1,4]	40 (16%)	1 (0.4%)	5 (6%)	<0.001[a*]<br>0.001[b*]<br><0.05[c*]
pH a. umbilicalis<7.00[1,4]	4 (2%)	0	0	<0.05[a*]<br>0.2[c]
Apgar<7 at 5 min[1,4]	9 (4%)	0	0	<0.01[a*]<br>0.08[c]

*Statistically significant at p-value <0.05. *a: VAD vs. SVD, b: SSCD vs. SVD, c: VAD vs. SSCD.* [1]n (%), [2]mean± sd, [3]median (min-max), [4]Chi^2 test, [5]Student T-test, [6]Wilcoxon rank sum test. BMI: body mass index, OFHR: ominous foetal heart rate, OAP: occipital-anterior position, NICU: neonatal intensive care unit. VAD: vacuum-assisted delivery, SVD: spontaneous vaginal delivery, SSCD: second stage caesarean delivery.

Confidential: For Review Only

Out of all VADs, 13% failed (n =33) and were delivered by subsequent delivery modes (forceps n=8, SSCD n=17 and forceps plus SSCD n=8). The failed VADs were included in the VAD group.

The Five-to-Fifteen questionnaire

A basic characteristics comparison between children with obvious difficulties in one or more domains according to the answers in the FTF (≥90th percentile) and children without difficulties (<90th percentile), according to normative data, was performed for each delivery mode. For all delivery modes, there were statistically significantly more women with a post-high school education in the group with children scoring <90th percentile. Apart from this, there were more subjectively heavy extractions among children scoring ≥90th percentile born after VAD (p-value <0.05). In the SSCD group there were more males among children scoring <90th percentile (p-value <0.01). Table 2 presents the age distribution of the children delivered by each delivery mode. The FTF was assessed in 2017 in the VAD group, in 2020 in the SVD group, and in 2019-2020 in the SSCD group.

Table 2: Age distribution among the children for each delivery mode

Age (years)	VAD (n =253)	SVD (n =247)	SSCD (n =86)
6y	-	-	11 (13%)
7y	50 (20%)	62 (25%)	13 (15%)
8y	82 (32%)	88 (36%)	16 (19%)
9y	104 (41%)	81(33%)	8 (9%)
10y	17 (7%)	16 (6%)	12 (14%)
11y	-	-	8 (9%)
12y	-	-	17 (20%)
13y	-	-	1 (1%)
Mean age at FTF completion	8 (±0.9)	8 (±0.9)	9 (±2.2)

N (%). VAD: vacuum-assisted delivery, SVD: spontaneous vaginal delivery, SSCD: second stage caesarean delivery, FTF: Five-to-Fifteen Questionnaire

The univariate regression analyses are presented in Table 3. There was a higher proportion of children scoring to have problems linked to motor skills and perception in the VAD group compared to the SVD group. The same results were seen when comparing SSCD to SVD. There were no calculated differences between the VAD and SSCD groups. In the multivariable logistic regression analysis the calculated aOR remained increased for problems with motor skills in children delivered with a VAD or SSCD compared to children delivered with a SVD.

Table 3: Number and percentages of children scoring ≥90th percentile in the FTF per main domain; a univariate and multivariable logistic regression analysis

Main domain	VAD n/total (%)	SVD n/total (%)	SSCD n/total (%)	OR (95% CI)	aOR (95% CI)
Motor skills	47/253 (19%)	23/246 (9%)	22/86 (26%)	2.2 (1.3- 3.8)[a]<br>3.3 (1.8-6.4)[b]<br>0.7 (0.4-1.2)[c]	2.2 (1.1- 4,7)[a]<br>3.2 (1.3-7.5)[b]<br>0.6 (0.3-1.1)[c]

Confidential: For Review Only

Executive functions	40/253 (16%)	34/247 (14%)	10/86 (12%)	1.2 (0.7-1.9)[a]<br>0.8 (0.4-1.8)[b]<br>0.8 (0.4-1.8)[c]	1.5 (0.8-3.1)[a]<br>1.2 (0.5-3.2)[b]<br>1.2 (0.5-2.5)[c]
Perception	42/253 (17%)	26/247 (11%)	18/86 (21%)	1.7 (1.002-2.9)[a]<br>2.3 (1.2-4.4)[b]<br>0.8 (0.4-1.4)[c]	1.9 (0.9-4.1)[a]<br>1.9 (0.8-4.7)[b]<br>0.6 (0.3-1.2)[c]
Memory	29/253 (11%)	26/247 (11%)	5/86 (6%)	1.1 (0.6-1.9)[a]<br>0.5 (0.2-1.4)[b]<br>2.1 (0.8-5.6)[c]	1.1 (0.5-2.4)[a]<br>0.5 (0.1-1.6)[b]<br>1.9 (0.7-5.1)[c]
Language	31/253 (12%)	22/247 (9%)	6/86 (7%)	1.4 (0.8-2.5)[a]<br>0.8 (0.3-2)[b]<br>1.9 (0.8-4.6)[c]	1.7 (0.7-3.8)[a]<br>0.6 (0.2-1.8)[b]<br>1.7 (0.7-4.2)[c]
Learning	30/195 (15%)	23/192 (12%)	7/62 (11%)	1.3 (0.8-2.4)[a]<br>0.9 (0.4-2.3)[b]<br>1.4 (0.6-3.4)[c]	1.6 (0.7-3.5)[a]<br>0.7 (0.2-2.2)[b]<br>1.2 (0.7-3.1)[c]
Social skills	30/252 (12%)	19/246 (8%)	6/81 (7%)	1.6 (0.9-3)[a]<br>0.9 (0.4-2.5)[b]<br>1.7 (0.7-4.2)[c]	1.2 (0.5-2.6)[a]<br>0.6 (0.2-1.9)[b]<br>1.6 (0.6-4.1)[c]
Emotional/ Behavioural problems	45/253 (18%)	29/247 (12%)	13/86 (15%)	1.6 (0.9-2.7)[a]<br>1.3 (0.7-2.7)[b]<br>1.2 (0.6-2.4)[c]	1.4 (0.7-2.8)[a]<br>1.4 (0.5-3.4)[b]<br>1.1 (0.6-2.2)[c]

a: VAD vs. SVD, b: SSCD vs. SVD, c: VAD vs. SSCD. FTF: Five-to-Fifteen questionnaire. VAD: vacuum-assisted delivery, SVD: spontaneous vaginal delivery, SSCD: second stage caesarean delivery. OR: odds ratio. The multivariable logistic regression analysis shows adjusted OR for parity, maternal educational level, gestational length, oxytocin usage in VAD vs. SVD and SSCD vs. SVD, and indication OFHR in VAD vs. SSCD. FTF: Five-to-Fifteen questionnaire. VAD: vacuum-assisted delivery, SVD: spontaneous vaginal delivery, SSCD: second stage caesarean delivery

There were significantly more children with obvious difficulties in two or more domains in the VAD and SSCD groups compared to the SVD group (Table 4).

Table 4: Number and percentages of children scoring ≥90th percentile in the FTF in ≥2 domains; a univariate logistic regression analysis

	**VAD** n/total (%)	**SVD** n/total (%)	**SSCD** n/total (%)	**OR (95%CI)**
≥2 domains	65/87 (75%)	37/73 (49%)	22/31 (71%)	1.96 (1.3-3.1)[a]<br>1.95 (1.1-3.6)[b]<br>1.00 (0.6-1.8)[c]

*Statistically significant at p-value<0.05. *a: VAD vs. SVD b: SSCD vs. SVD c: VAD vs. SSCD.* FTF: Five-to-Fifteen questionnaire, VAD: vacuum-assisted, SVD: spontaneous vaginal delivery, SSCD: second stage caesarean delivery

The incidence of the diagnoses acquired from the SNBHW are presented in Table 5. There are more children with an attention deficit/hyperactivity disorder delivered with VAD compared to SVD (p-value <0.01) and to ECD (p-value <0.05). Furthermore, children born by VAD had a

Confidential: For Review Only

higher incidence of neurodevelopmental diagnoses than children born by SVD (p-value <0.001).

Table 5 Children´s diagnoses according to ICD-10

	VAD (n =253)	SVD (n =247)	SSCD (n =86)
Communication disorders (F80)	4 (2%)	1 (0.4%)	0
Specific developmental disorder of motor function (F829)	2 (0.8%)	0	0
Autism spectrum disorders (F84)	12 (5%)	6 (2%)	0
Unspecified disorder of psychological development (F899)	0	0	1 (1%)
Attention deficit hyperactivity disorder (F90)	23 (9%)	8 (3%)	1 (1%)
Oppositional defiant disorder (F913)	1 (0.4%)	0	0
Tic disorders (F95)	2 (1%)	0	0
Enuresis (F980)	4 (2%)	4 (2%)	1 (1%)
Encopresis (F981)	1 (0.4%)	0	3 (3%)
Developmental and emotional disorders (F98)	2 (0.8%)	0	1 (1%)
Unspecified mental disorder (F999)	3 (1%)	2 (0.8%)	0
Epilepsy (G40)	1 (0.4%)	2 (0.8%)	1 (1%)
Neurodevelopmental diagnosis	36 (14%)	15 (6%)	7 (8%)
Child with ≥2 neurodevelopmental diagnoses	20 (8%)	6 (2%)	2 (2%)

N (%). a*: VAD vs. SVD, b: SSCD vs. SVD, c: VAD vs. SSCD.* VAD: vacuum-assisted delivery, SVD: spontaneous vaginal delivery, SSCD: second stage caesarean delivery.

Confidential: For Review Only

Discussion

Main findings

According to the results from the studied cohort, low- and mid-station VADs imply increased odds of long-term neurodevelopmental difficulties in motor skills and perception between seven and ten years of age compared to spontaneously delivered children. Similar findings were seen in the group delivered with an SSCD. After adjusting for the proposed confounding variables, the increased odds for difficulties in motor skills after a VAD or an SSCD was maintained. The incidence of attention deficit hyperactivity disorder (ADHD) was higher among children born after a VAD.

Considering the relatively frequent use of VAD in modern obstetrics, it is striking how little is known about the long-term outcomes. According to the current study, this group may be at risk for neurodevelopmental difficulties. The information available does not allow one to determine whether the VAD procedure causes these difficulties, or whether the entire delivery process contributes, since children born after an SSCD were calculated to be at a similar risk. It may be that the complete situation, including the lack of progress in the second stage of labour, and the maternal and foetal conditions, are responsible for the problematic outcome. It appears, however, that children born after VAD might have an increased risk of ADHD, as indicated by the register data. This increased incidence was not observed in the SSCD group.

Strengths and limitations

This is a study with a well-defined cohort of children, investigating long-term outcomes using a validated screening method. By including the registered neurodevelopmental ICD-10 diagnoses at the SNBHW, the value of the study is enhanced.

Even though the validated questionnaire contains a normative reference group, we included a control group with children born via SVD to increase validity. In addition, SSCD group with a foetal head station at level 0 or more, without a prior VAD attempt, is a clinically relevant comparison group. This group had a significantly longer duration of the second stage (a median of five hours) and showed similar results in the FTF as the VAD group. On the other hand, there were more children with neurodevelopmental diagnoses and NICU admissions in the VAD group.

It might be questioned whether parents' estimates of a child's development are objective. However, the FTF is not a diagnostic tool, and a multi-professional assessment is needed to diagnose neurodevelopmental disorders in children and youth. Since the questionnaire was distributed only in the Swedish language, an inevitable selection of parents might exist, but this applies to the whole cohort.

The maternal educational level was included as a proxy for socioeconomic status, since most questionnaires were filled out by mothers. Mothers with higher educational levels more often had children scoring <90th percentile, and mothers in the VAD group had significantly lower educational level than the other two groups. This variable was adjusted for in the multivariable regression analysis.

The low response rate in the SVD group is a limitation and could be attributed to the fact that a control group is less likely to respond. This could potentially lead to selection bias, resulting in parents of children with difficulties being more likely to respond. Alternatively, it may be that parents who experienced a traumatic delivery were less prone to respond. The response analysis in the VAD group did not reveal any differences, except a higher rate of mid-station VADs in the non-respondent group, which is believed to not affect the result, except a possible risk for underestimation of FTF scores.

Including the failed VADs in this group may bias the result by including children with worse outcomes but shows the relevance in the clinical reality. On the other hand, the SSCD group was clinically more uniform for comparison. Since the SSCD group is smaller, differences may be hidden, and conclusive insight is hard to draw regarding registered diagnosis due to sparse data. To remedy the small sample size in the SSCD group, the age range was extended while still suitable for the FTF. We believe this was necessary to try to answer the research question without introducing risks.

Interpretation

Confidential: For Review Only

The study is observationally designed to screen for neurodevelopmental difficulties rather than to reveal causal relationships. With the use of FTF several neurodevelopmental aspects were possible to study. The results are in contrast with the few existing studies reporting on specific long–term neurodevelopmental aspects,[19-21, 23, 33] but these have not stratified according to the VAD type, they have smaller cohorts, and did not use validated follow–up methods.

Even though children had motor skill problems according to the FTF, they were not registered with individual motor skill diagnoses. This may rather be revealed in a more complex form i.e., as a comorbidity in ADHD. Whether or not low– and mid-station station VAD or SSCD affect specific parts of the foetal brain resulting in impaired neurodevelopment for different skills, is unknown. Although the etiology of diagnoses such as ADHD is heterogeneous and unclear, it is known to be associated with traumatic brain injury.[34] Apart from this, there is an established association between ADHD and heredity.[35, 36] Registered neurodevelopmental diagnoses among parents or siblings were not collected.

Conclusion

Obstructed labour at the second stage in term deliveries is a delicate mission for clinicians. Although interpreted with caution, the significant difference in long-term neurodevelopmental difficulties in children delivered with VAD or SSCD compared to a SVD is worrying and indicates the need for increased knowledge in the field. If additional studies show similar results, it seems necessary to revise the existing guidelines for VAD and SSCD.

Ethical approval Ethical approval was obtained by the Regional Ethical Review Board Stockholm, Sweden (DNR 2014/1860–31, DNR 2017/1411–32, DNR 2019/04880 and DNR 2021/02457).
Funding statement This work was supported by the Centre for Innovative Medicine (grant number FoUI-954462), Karolinska Institutet, the General Maternity Hospital Foundation (grant number N/A), and Kommunfullmäktige, Stockholm Stad, grant number FoUI-953029 and FoUI-955070).
Competing interest statement All authors have completed the ICMJE uniform disclosure form at http://www.icmje.org/disclosure-of-interest/ and declare: no support from any organisation for the submitted work; no financial relationships with any organisations that might have an interest in the submitted work in the previous three years; no other relationships or activities that could appear to have influenced the submitted work.
Authors' contributions GA and MW designed the study, acquired, analysed, interpreted the data, and wrote and revised the manuscript. SR acquired, analysed, interpreted the data, and wrote and revised the manuscript. KL analysed, interpreted the data, and revised the manuscript. The corresponding author attests that all listed authors meet authorship criteria and that no others meeting the criteria have been omitted.
Acknowledgements The paper is part of Dr. Romero's doctoral thesis.
STROBE checklist see supplementary files
Data sharing statement "The Corresponding Author has the right to grant on behalf of all authors and does grant on behalf of all authors, if the manuscript is accepted, a worldwide licence to the Publishers and its licensees in perpetuity, in all forms, formats and media (whether known now or created in the future), to i) publish, reproduce, distribute, display and store the Contribution, ii) translate the Contribution into other languages, create adaptations, reprints, include within collections and create summaries, extracts and/or, abstracts of the Contribution, iii) create any other derivative work(s) based on the Contribution, iv) to exploit all subsidiary rights in the Contribution, v) the inclusion of electronic links from the Contribution to third

Confidential: For Review Only

party material wherever it may be located; and, vi) licence any third party to do any or all of the above."

Transparency declaration: The lead author (GA) affirms that the manuscript is an honest, accurate, and transparent account of the study being reported; that no important aspects of the study have been omitted and that any discrepancies from the study as planned have been explained.

Confidential: For Review Only

References

1. Malmstrom, T., *Vacuum extractor, an obstetrical instrument.* Acta Obstet Gynecol Scand Suppl, 1954. **33**(4): p. 1-31.
2. Martin Ja Fau - Hamilton, B.E., et al., *Births: Final Data for 2017.* (1551-8922 (Print)).
3. The Swedish National Board of Health and Welfare, T. *Statistikbas för graviditeter, förlossningar och nyfödda.* 2020; Available from: https://www.socialstyrelsen.se/statistik-och-data/statistik/alla-statistikamnen/graviditeter-forlossningar-och-nyfodda/.
4. NHS, *NHS Maternity Statistics, England*. 2021, NHS Digital: England.
5. Canadian Institute for Health Information, *Assisted delivery rate (overall) among vaginal deliveries*. 2022.
6. (RANZCOG), T.R.N.Z.C.o.O.a.G., *Instrumental vaginal birth.* 2020: RANZCOG web page.
7. Macfarlane, A.J., et al., *Wide differences in mode of delivery within Europe: risk-stratified analyses of aggregated routine data from the Euro-Peristat study.* Bjog, 2016. **123**(4): p. 559-68.
8. Ekeus, C., U. Hogberg, and M. Norman, *Vacuum assisted birth and risk for cerebral complications in term newborn infants: a population-based cohort study.* BMC Pregnancy Childbirth, 2014. **14**: p. 36.
9. Muraca, G.M., et al., *Maternal and neonatal trauma following operative vaginal delivery.* CMAJ, 2022. **194**(1): p. E1-E12.
10. Gebremariam, A., *Subgaleal haemorrhage: risk factors and neurological and developmental outcome in survivors.* Ann Trop Paediatr, 1999. **19**(1): p. 45-50.
11. Towner, D.R. and M.C. Ciotti, *Operative vaginal delivery: a cause of birth injury or is it?* Clin Obstet Gynecol, 2007. **50**(3): p. 563-81.
12. Singh, A. and P. Rathore, *A comparative study of feto-maternal outcome in instrumental vaginal delivery.* J Obstet Gynaecol India, 2011. **61**(6): p. 663-6.

13. Hehir, M.P., et al., *Increasing rates of operative vaginal delivery across two decades: accompanying outcomes and instrument preferences.* Eur J Obstet Gynecol Reprod Biol, 2013. **171**(1): p. 40-3.
14. Muraca, G.M., et al., *Perinatal and maternal morbidity and mortality among term singletons following midcavity operative vaginal delivery versus caesarean delivery.* BJOG, 2018. **125**(6): p. 693-702.
15. Walsh, C.A., M. Robson, and F.M. McAuliffe, *Mode of delivery at term and adverse neonatal outcomes.* Obstet Gynecol, 2013. **121**(1): p. 122-8.
16. Halscott, T.L., et al., *Maternal and Neonatal Outcomes by Attempted Mode of Operative Delivery From a Low Station in the Second Stage of Labor.* Obstet Gynecol, 2015. **126**(6): p. 1265-72.
17. Vacca, A., *Reducing the Risks of a Vacuum Delivery.* Fetal Medicine Review, 2006. **17 (4)**: p. 301-315.
18. Murphy, D.J., et al., *Assisted Vaginal Birth: Green-top Guideline No. 26.* BJOG, 2020. **127**(9): p. e70-e112.
19. Ahlberg, M., C. Ekeus, and A. Hjern, *Birth by vacuum extraction delivery and school performance at 16 years of age.* Am J Obstet Gynecol, 2014. **210**(4): p. 361 e1-361 e8.
20. Ngan, H.Y., et al., *Long-term neurological sequelae following vacuum extractor delivery.* Aust N Z J Obstet Gynaecol, 1990. **30**(2): p. 111-4.
21. Johanson, R.B., et al., *Maternal and child health after assisted vaginal delivery: five-year follow up of a randomised controlled study comparing forceps and ventouse.* Br J Obstet Gynaecol, 1999. **106**(6): p. 544-9.
22. Bjerre, I. and K. Dahlin, *The long-term development of children delivered by vacuum extraction.* Dev Med Child Neurol, 1974. **16**(3): p. 378-81.
23. Seidman, D.S., et al., *Long-term effects of vacuum and forceps deliveries.* Lancet, 1991. **337**(8757): p. 1583-5.
24. Mastrolia, S.A., et al., *Failed Vacuum and the Long-Term Neurological Impact on the Offspring.* Am J Perinatol, 2017. **34**(13): p. 1306-1311.
25. Ayala, N.K., et al., *Operative vaginal delivery and third grade educational outcomes.* Am J Obstet Gynecol MFM, 2020. **2**(4): p. 100221.
26. ACOG, *Operative Vaginal Birth: ACOG Practice Bulletin, Number 219.* Obstet Gynecol, 2020. **135**(4): p. e149-e159.
27. Vladic-Stjernholm, Y.W.A.A.-W.I. *Instrumental delivery by vacuum extraction: advice for clinical recommendations (in Swedish): Löf*. 2017; Available from: https://lof.se/filer/Instrumentell-forlossning-med-sugklocka.pdf.
28. Kadesjo, B., et al., *The FTF (Five to Fifteen): the development of a parent questionnaire for the assessment of ADHD and comorbid conditions.* Eur Child Adolesc Psychiatry, 2004. **13 Suppl 3**: p. 3-13.
29. Trillingsgaard, A., et al., *Developmental profiles on the basis of the FTF (Five to Fifteen) questionnaire-clinical validity and utility of the FTF in a child psychiatric sample.* Eur Child Adolesc Psychiatry, 2004. **13 Suppl 3**: p. 39-63.
30. Korkman, M., et al., *Screening of developmental disorders in five-year-olds using the FTF (Five to Fifteen) questionnaire: a validation study.* Eur Child Adolesc Psychiatry, 2004. **13 Suppl 3**: p. 31-8.
31. Bohlin, G. and L.O. Janols, *Behavioural problems and psychiatric symptoms in 5-13 year-old Swedish children-a comparison of parent ratings on the FTF (Five to Fifteen)*

Confidential: For Review Only

with the ratings on CBCL (Child Behavior Checklist). Eur Child Adolesc Psychiatry, 2004. **13 Suppl 3**: p. 14-22.

32. Ludvigsson, J.F., et al., *External review and validation of the Swedish national inpatient register.* BMC Public Health, 2011. **11**: p. 450.
33. Nilsen, S.T., *Boys born by forceps and vacuum extraction examined at 18 years of age.* Acta Obstet Gynecol Scand, 1984. **63**(6): p. 549-54.
34. Asarnow, R.F., et al., *Association of Attention-Deficit/Hyperactivity Disorder Diagnoses With Pediatric Traumatic Brain Injury: A Meta-analysis.* JAMA Pediatr, 2021. **175**(10): p. 1009-1016.
35. Larsson, H., et al., *The heritability of clinically diagnosed attention deficit hyperactivity disorder across the lifespan.* Psychol Med, 2014. **44**(10): p. 2223-9.
36. Faraone, S.V. and H. Larsson, *Genetics of attention deficit hyperactivity disorder.* Mol Psychiatry, 2019. **24**(4): p. 562-575.

Confidential: For Review Only

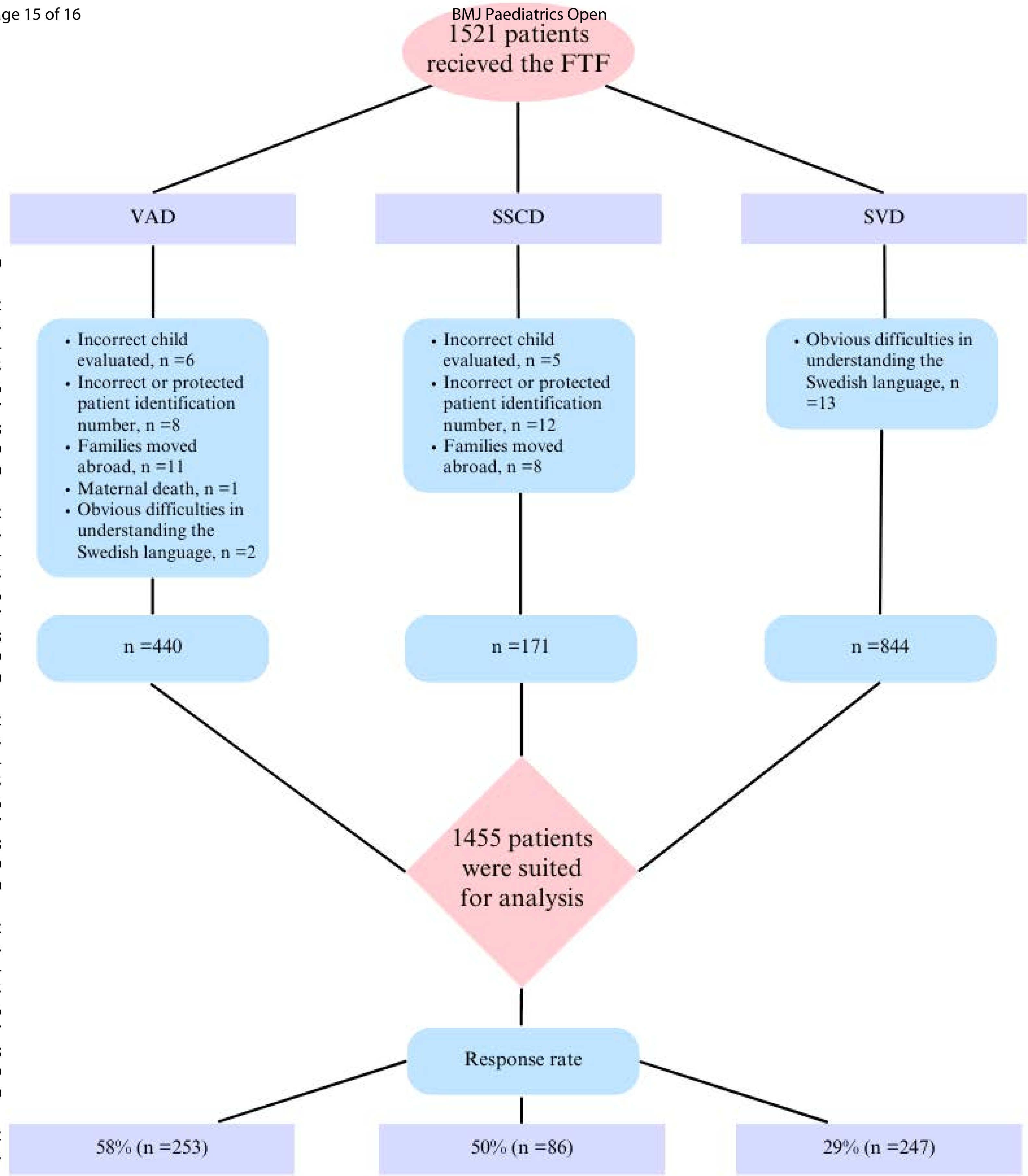

Figure 1: Study flowchart

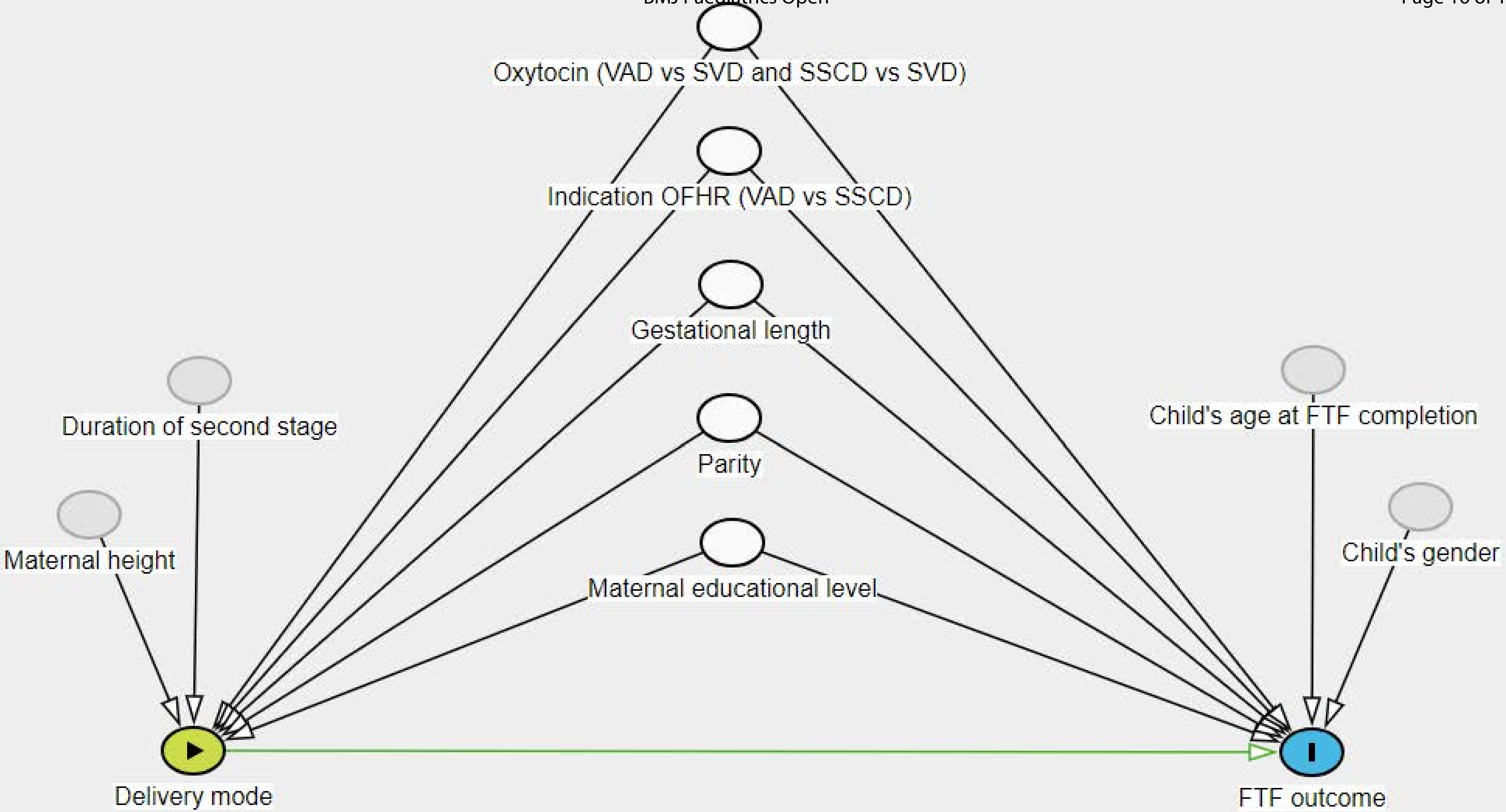

Figure S1: Directed Acyclic Graph (DAG)

Table S1: Descriptive analysis of respondents and non-respondents in the vacuum-assisted delivery group

	FTF (n =253)	No FTF (n =187)	p-value
**Maternal characteristics**			
BMI (kg/m^2)[3]	23 (16-50)[§]	23 (17-39)[†]	0.82[6]
Nulliparous[1]	208 (82%)	146 (78%)	0.28[4]
Previous caesarean[1]	23 (9%)	20 (11%)	0.58[4]
Gestational length, days[2]	281±8	282±9	0.30[5]
**Delivery characteristics**			
First stage of labour, hours[2]	9±5	9±5	0.56[5]
Second stage of labour, hours[2]	2±2	2±2	0.22[5]
Epidural[1]	175 (72%)	135 (78%)	0.22[4]
Oxytocin[1]	242 (96%)	172 (92%)	0.11[4]
Indication OFHR[1]	108 (43%)	89 (48%)	0.31[4]
Station Mid[1]	148 (59%)	133 (71%)	<0.05[4*]
Number of pulls, n[3]	4 (1-10)	4 (1-13)	0.4[6]
Time during vacuum extraction, min[3]	7 (1-31)	7 (1-29)	0.94[6]
Position OAP[1]	221 (87%)	159 (85%)	0.48[4]
Subjectively heavy extraction[1]	58 (25%)	48 (28%)	0.44[4]
Failed vacuum extraction[1]	34 (13%)	33 (18%)	0.23[4]
Cup detachment[1]	39 (15%)	29 (16%)	0.98[4]
Shoulder dystocia[1]	11 (4%)	13 (7%)	0.23[4]
**Perinatal characteristics**			
Birth weight, g[2]	3668 ± 447	3641 ± 455	0.54[5]
Gender male[1]	138 (55%)	118 (63%)	0.07[4]
pH<7.00[1]	4 (2%)[‡]	0[⸸]	0.09[4]
Apgar<7 at 5 min[1]	9 (4%)	9 (5%)	0.51[4]
Severe outcome[1] (ICH, HIE, seizures, subgaleal haematoma)	4 (2%)	5 (3%)	0.42[4]

*Statistically significant at p-value <0.05. [1]n (%), [2]mean±sd, [3]median (min–max), [4]Chi^2 test, [5]Student T-test, [6]Wilcoxon rank sum test. [§]missing data 11%, [†]missing data 10%, [‡]missing data 18%, [⸸]missing data 19%. FTF: Five- to Fifteen questionnaire. BMI: body mass index, OFHR: ominous foetal heart rate, OAP: occipital-anterior position, ICH: intracranial haemorrhage, HIE: hypoxic ischemic encephalopathy

Confidential: For Review Only
